# Nursing care recommendation for pediatric COVID-19 patients in the hospital setting: A brief scoping review

**DOI:** 10.1371/journal.pone.0263267

**Published:** 2022-02-03

**Authors:** Defi Efendi, Faizul Hasan, Regina Natalia, Ayuni Rizka Utami, Ismaila Sonko, Titik Ambar Asmarini, Risna Yuningsih, Dessie Wanda, Dian Sari

**Affiliations:** 1 Department of Pediatric Nursing, Faculty of Nursing, Universitas Indonesia, Depok, Indonesia; 2 Neonatal Intensive Care Unit, Universitas Indonesia Hospital, Depok, Indonesia; 3 School of Nursing, Taipei Medical University, Taipei, Taiwan; 4 School of Nursing, Mitra Bunda Health Institute, Batam, Indonesia; 5 Ministry of Health and Social Welfare, The Quadrangle, Banjul, The Gambia, West Africa; 6 Neonatal and Pediatric Intensive Care Unit, Gatot Soebroto Indonesian Central of Army Hospital, Jakarta, Indonesia; 7 Neonatal Intensive Care Unit, Dr. Dradjat Prawiranegara General Hospital, Banten, Indonesia; 8 School of Nursing, Prima Nusantara Health Institute, Bukittinggi, Indonesia; Heidelberg University Hospital, GERMANY

## Abstract

**Background:**

The hospitalization of children during the COVID-19 pandemic has affected their physical and mental health. Pediatric nurses have faced challenges in providing high-quality nursing care for children and their families. However, the pediatric nursing care recommendations for COVID-19 patients in the hospital setting remain unclear. The current scoping review provides recommendations for nursing interventions for pediatric COVID-19 patients in the hospital setting.

**Methods and findings:**

The selected articles containing management and nursing recommendations for COVID-19 that have occurred in pediatric patients ages 0–19 years old. A search strategy was developed and implemented in seven databases. We included peer-reviewed articles that reported observational or interventional studies, as well as policy papers, guides or guidelines, letters and editorials, and web articles. A total of 134 articles and other documents relevant to this review were included. We categorized the results based on The Nursing Intervention Classification (NIC) taxonomy which consists of six domains (e.g., Physiological: Basic); eleven classes (e.g., Nutrition Support); and eighteen intervention themes (e.g., Positioning, Family Presence Facilitation, Family Support, and Discharge Planning).

**Conclusion:**

Apart from the intervention of physical problems, there is a need to promote patient- and family-centered care, play therapy, and discharge planning to help children and families cope with their new situation.

## Introduction

Corona virus disease is defined as an infectious disease caused by the newly discovered coronavirus SARS-CoV-2 [1–3]. It was identified in Wuhan, China, on 29 December 2019, as reported by the World Health Organization [4]. This disease was officially named COVID-19 by the WHO on 11 February 2020 [5]. To date, its spread continues to increase worldwide [6]. On 16 November 2021, an estimated 253,640,693 confirmed cases and 5,104,899 deaths caused by COVID-19 had been reported globally [7]. Most cases have occurred in the Americas, Europe, and South-East Asia [7].

Children are part of a population that requires further study [8]. A systematic literature review of 23 countries estimated that the prevalence of children suffering from COVID-19 was 1.9% [9]. Data reported from Spain showed 41 confirmed cases in children, of which 60% required hospital care and 9.7% needed to be admitted to the Pediatric Intensive Care Unit (PICU) [10]. Dong, et al. revealed that 2,143 pediatric patients (731 confirmed and 1412 suspected) who were admitted to hospital developed into moderate to severe cases, and 40% and 0.6% required admission to a Pediatric Intensive Care Unit (PICU), respectively [11]. In the USA, the CDC reported 2575 cases among children, of which up to 20% required hospital admission, and 2% needed care in a PICU [12]. Most studies in the literature reported physical complaints, such as fever, cough, rhinorrhea, nasal congestion, myalgia, fatigue, sore throat, shortness of breath, dyspnea, abdominal pain, diarrhea, vomiting, nausea, headache, mild to severe respiratory distress, and Multisystem Inflammatory Syndrome in Children (MIS-C) [13–23]. Moreover, an extensive systematic review concluded that the symptoms of COVID-19 in children differed widely from adult cases [24]. Nursing care is designed to overcome problems related to specific symptoms and conditions of the patient. However, the number of studies conducted on children with COVID-19 is limited [25].

In addition to physical complaints, the hospitalization itself can be a harmful and stressful experience for children [26]. Hospitalized children expressed fears, uncertainty, anger, helplessness, and anxiety caused by several medical procedures due to a lack of information and an unfamiliar physical and social environment [27–29], which could affect the mental and spiritual health of the child and parent [30]. A similar negative impact of hospitalization in the pediatric COVID-19 context has also been reported [31–33]. Moreover, restricted physical movement, parental separation, stigma, and social exclusion caused by hospitalized quarantined protocol were shown to complicate the care of pediatric patients with COVID-19 [32, 34–36]. Because of such issues, it has been particularly challenging for pediatric nurses to provide nursing care during the COVID-19 pandemic across various limitations [37].

Several nursing care guidelines have been developed to support nurses in managing COVID-19 patients in a wide range of situations across the world, such as initial general nursing care [38, 39], palliative care guidance for nursing homes [40], oncology nursing [41], and emergency nursing [42]. However, to our knowledge, no comprehensive nursing care guidelines have been developed to address the specific needs of children suffering from COVID-19 in a hospital setting. Hence, providing clear clinical nursing management guidance for nursing pediatric patients with COVID-19 is urgently needed. This study aims to provide guidance on nursing care for pediatric COVID-19 patients based on a systematic review of the relevant literature.

## Methods

### Study design

Arksey and O’Malley’s [43] framework, which is a commonly used guide in conducting a scoping review [44], consists of five steps: 1) identifying a clear research objective and search strategies; 2) identifying relevant research articles; 3) selecting research articles; 4) extracting and charting data; and 5) summarizing, discussing, analyzing, and reporting the results [45]. In this current scoping review, the Joanna Briggs Institute (JBI) guidelines, which is a leading source of scoping review guidance, was used as the primary protocol for conducting this research [46]. JBI guidelines was an extended and refined work from Arksey and O’Malley’s framework.

### Literature search strategy

A systematic search of the CINAHL, Science Direct, ProQuest, Embase, SpringerLink, PubMed, and Taylor and Francis databases was performed, including references from inception of databases to 10 February 2021, using the terms ‘children’ AND ‘COVID-19’ with a combination of keywords. A detailed example of the search strategy is provided in S1 Table.

### Identification and selection of relevant studies

Studies were eligible for a full-text analysis when all patients were under 18 years and the publication related to in an outpatient or in-hospital environment. Randomized controlled trials, controlled and non-controlled before-and-after studies, controlled and non-controlled interrupted time series, and cohort studies were included. The overall criteria of this study are provided in S2 Table.

**Fig 1:**
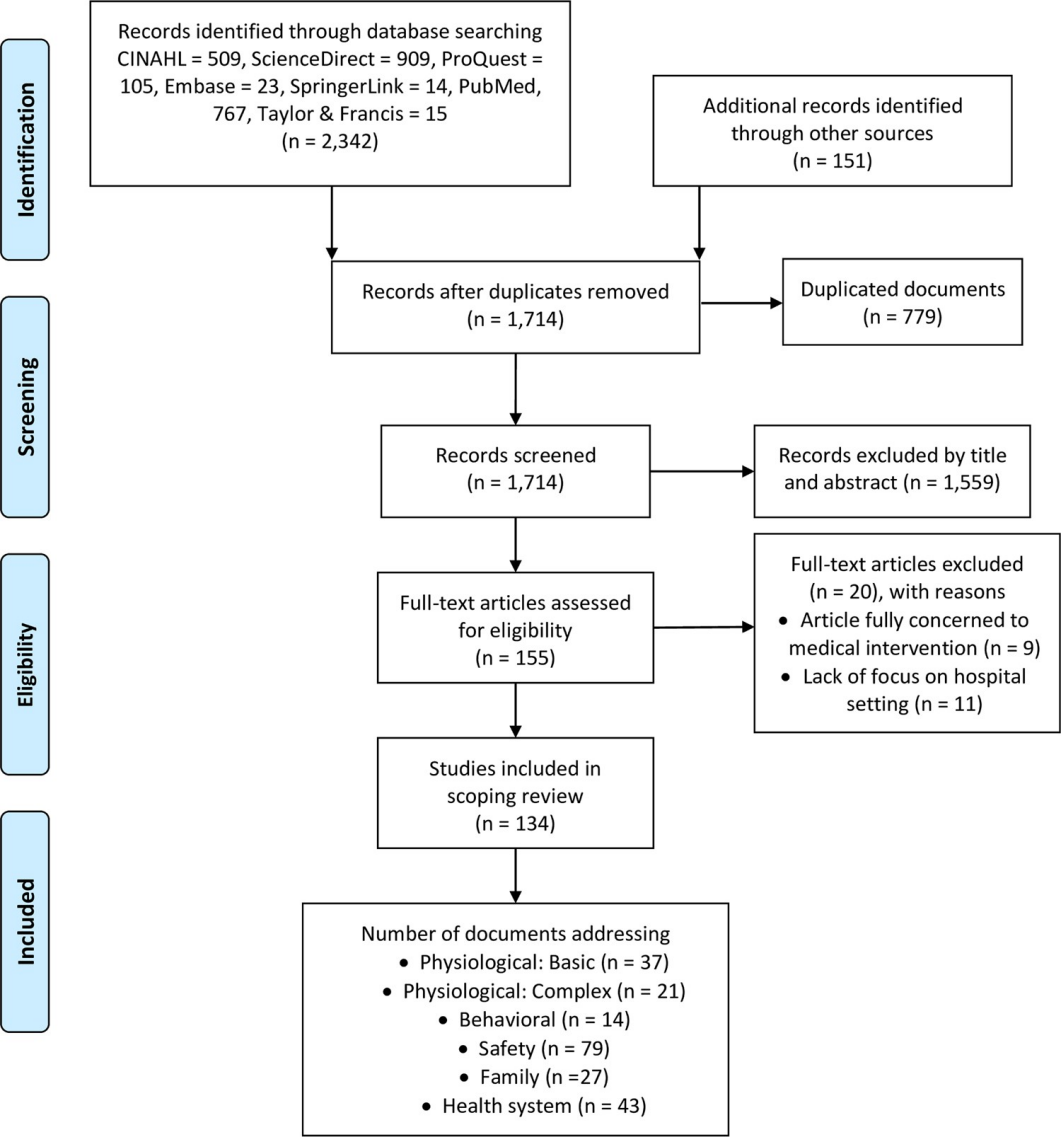
PRISMA flow diagram for the scoping review process.

Systematic reviews, scoping reviews, and meta-analyses were excluded. Studies on both adults and children from which the extraction of pediatric data was not possible were also excluded. We excluded studies published before December 2020 because the concept of COVID-19 was formally introduced in that year. We did not include articles about types of disease other than COVID-19 and its’ related medical condition similarly MIS-C. Fig 1 shows the process used to search and select the research articles. Four investigators independently performed reviews of the titles, abstracts, and full texts (A.R.U., R.N., T.A.A., and R.Y.). Any disagreement regarding the collection of studies was resolved by discussion with a fifth reviewer (D.E.).

### Data synthesis and analysis

A structured data collection was conducted and a table (S3 Table) was created to document extracted data that summarized information about authors, year of publication, country, study time, type of article, and purpose of the study. Five authors (A.R.U., R.N., T.A.A., R.Y., and D.S.) conducted the data extraction from the included studies. They read every full text thoroughly to identify any statement that could be transformed into a clinical nursing intervention in the hospital setting. A.R.U., and R.N. adopted the Nursing Intervention Classification (NIC, 7th ed.) to rearrange the extracted data into suitable domains, classes, and interventions [47]. Each included study has chance to be generated in more than one domain, class, and intervention. Because it is a specialty among other pediatric patients, we divided nursing interventions for neonates into separate parts in each table. The recommendations were categorized according to the appropriate class and intervention. D.E. then performed an in-depth review for a new set of nursing recommendations that was derived from the previous authors who extracted the data (A.R.U., R.N., T.A.A., R.Y., and D.S.). Any changes made by D.E. were discussed with all authors involved in the data analysis process. We used our expertise and experience in delivering nursing care in the pediatric and neonatal wards, as well as in intensive care, to ensure the fine-tuning and applicability of these recommendations. We reported this scoping review following the PRISMA-ScR Checklist (S1 Checklist) [48].

## Results

**Fig 2:**
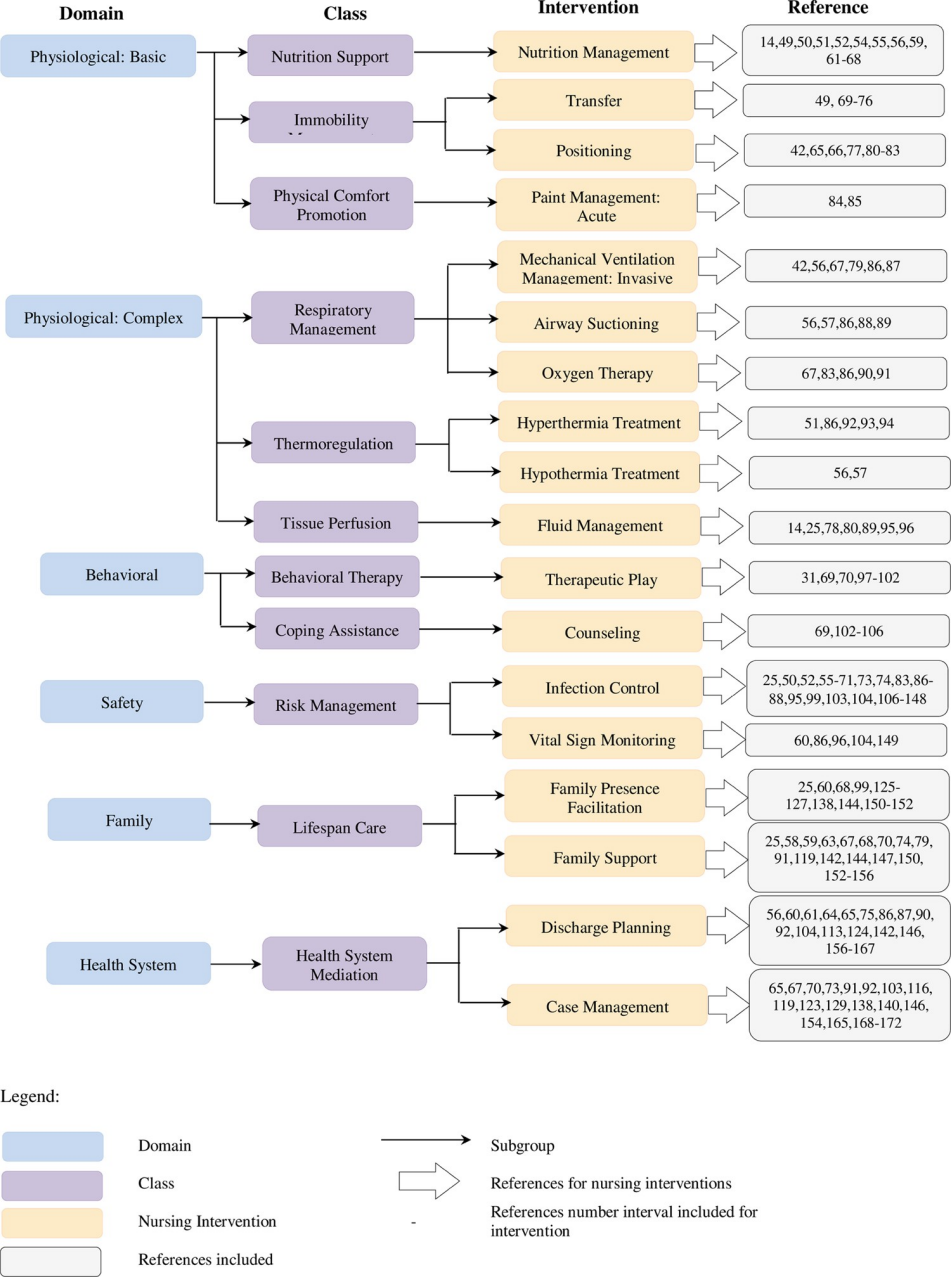
NIC framework for including study.

We initially identified 2,493 articles and screened the abstracts and titles for inclusion (Fig 1). After the removal of duplicates and irrelevant studies, 134 full-text articles were included in the review. The characteristics of the included studies are listed in S3 Table. The extracted articles contained information on child care recommendations in health care settings during the COVID-19 pandemic using a framework based on the NIC taxonomy [47]. Accordingly, from the documents included, we disseminate them into the eligible taxonomy comprise: six domains (Physiological: Basic, Physiological: Complex, Behavioural, Safety, Family, and Health System), eleven classes (Nutrition Support, Immobility Management, Physical Comfort Promotion, Respiratory Management, Thermoregulation, Tissue perfusion, Behavioural Therapy, Coping Assistance, Risk Management, Lifespan Care, Health System Mediation), and eighteen intervention themes (Nutrition Management, Transfer, Positioning, Pain Management: Acute, Mechanical Ventilation, Management: Invasive, Airway Suctioning, Oxygen Therapy, Hyperthermia Treatment, Hypothermia Treatment, Fluid Management, Therapeutic Play, Counseling, Infection Control, Family Presence Facilitation, Family Support, Discharge Planning, Case Management). The distribution of eligible studies in the taxonomy describes in Fig 2.

Among the six domains, the Physiological: Basic domain was widely discussed among the variety of interventions for Nutrition Support, Immobility Management, and Physical Comfort Promotion. Table 1 provides 16 interventions for Nutrition Management, Transfer, Positioning, and Pain Management: Acute. In addition, the Physiological: Complex domain showed various intervention recommendations for classes of Respiratory Management, Thermoregulation, and Tissue Perfusion (as described in Table 1). Thirteen interventions were identified: Mechanical Ventilation, Airway Suctioning, Oxygen Therapy, Hyperthermia Treatment, Hypothermia Treatment, and Fluid Management.

**Table 1:** Physiological basic and complex domain.

Domain	Class	Intervention	Summary of Intervention
(1) Physiological: Basic	Nutrition Support	Nutrition Management	• Ensure adequate fluid, and nutrient intake [14, 49, 50]. Fulfil energy supply of 25–30 kcal /kg/day [51] and pay attention to the nutrition strategy for PICU critical children [52], post-critical recovery, and the acute phase [52–54]. **NEONATES** • Rooming-in, and direct breastfeeding or expressing breast milk (if the baby is sick and not admitted to rooming-in) is recommended for babies of mothers who have no symptoms / mild symptoms of COVID-19 and mothers can care for LBW independently by controlling the spread of infection [49, 55–64]• Caregivers who are not infected can assist in giving breast milk to the baby [61, 65] using a special bottle [62].• Give the mother support to breastfeeding the baby [56] and make decisions about breastfeeding [63, 66] by discussing the risks, and benefits of breastfeeding the baby in the covid-19 positive mother [67].• Assess the possibility of breastfeeding with re-lactation, wet nursing (another woman breastfeeding the baby), or donor breast milk according to culture and patient acceptance [66]. • Use pasteurized donor breast milk, and breastfeeding donors have performed a blood test if expressed breast milk is not available [62, 68]. Another suggestion states that dairy milk should not be pasteurized because it is believed that it is not a carrier of infection and pasteurization can reduce the biological and immunological levels of breast milk [59]. • Stop breastfeeding donations for two weeks if the mother is suspected of COVID-19 until she gets two negative swabs [62].
	Immobility Management	Transfer	• Use special vehicles to transfer infected patients, strict protection for transportation staff, vehicle disinfection [49, 69, 70], and pay attention to patient transfer procedures [71]. • Pay attention to the transport of the intubated patient. patient using ventilator needs to be supplemented with viral filters and consideration of additional sedation [72]. **NEONATES** • Transportation of neonates with suspected/confirmed COVID-19 must be equipped with PPE, disinfectant, ventilator, vital sign monitor, first aid drugs, and a closed incubator for transfer equipment [73–75]. • It is recommended to use a NETS ambulance as COVID-19 NETS to transport newborns [76].
		Positioning	• Position the patient in an early prone position or prolonged moderate to severe PARDS (i.e. PaO_2_ / FiO_2_ <150; OI ≥ 12; OSI ≥ 10 for 12–18 hours per day (avoid disconnection) [42, 65, 77–81] and discontinue if PaO_2_ / FiO_2_ ≥ 150; OI <12; OSI <10 [66, 82] or change the position to supine during a heart attack [80]. If the patient is intubated, cardiopulmonary resuscitation (CPR) can remain prone [80]. • If the long duration of prone position is not achievable, try to position the patient 1–2 hours 3–4 times/day [77, 83].
	Physical Comfort Promotion	Pain Management: Acute	• Treatments in pain management include physical therapy, psychology, medical providers, and complementary services such as massage, and acupuncture [84]. • Perform non-pharmacological techniques according to the location of the pain. Provide psychotherapy, such as distraction, spending time with family, playing games with others, laughter. Provide physical therapy such as acute pain, preferably rest but chronic pain, need regular movement every day so that much pain is reduced, for example; take a walk around the neighbourhood with the family, do fun online dance or yoga classes, ride a bike. Relaxation skills: diaphragmatic breathing, progressive muscle relaxation, guided imagery [84]. **NENEONATES** • Give D10% as a non-pharmacological action to reduce pain due to invasive action in neonates [85].
(2) Physiological: Complex	Respiratory Management	Mechanical Ventilation Management: Invasive	• Use cuffed endotracheal tubes [67, 79], and monitor the ETT cuff pressure every 6–12 hours to ensure there is no leak, and the pressure is at the safe limit of <20cm H_2_O [42]. • Use a disposable ventilator circuit for each new patient [86], attach a viral filter (HEPA) to the expiration circuit [56, 86] or a hydrophobic mechanical filter attached to the inspirational and expiratory section [87]. Heat and moisture exchanger (HME) must be replaced every 48 hours or when dirty [86].
		Airway Suctioning	• Closed suction is recommended [86, 88] if there is airway obstruction [89] or only if needed [57] and there are indications [56, 86].
		Oxygen Therapy	• Maintain SpO_2_> 92% [90], pay attention to the oxygen saturation value that is safe for patients [83]. • Early intubation is recommended for children with severe/critical symptoms of COVID-19 rather than non-invasive ventilation or high flow nasal cannula because it can produce aerosols [86] which should be minimized [91]. • Provide adequate humidity, and heating when giving oxygen, especially at concentrations> 3–4 L / minute [83]. • Avoid using high flow nasal cannulas [67], and NIV considering the potential for aerosol formation [86].
	Thermoregulation	Hyperthermia Treatment	• Observe body temperature regularly. It needs serious attention related to temperature increase, even though the increase is not too high [92]. • Pediatric patients with high fever exceeding 38.5°C and seem uncomfortable, it is recommended to physically cool down with warms baths, use antipyretic patches or fever-reducing drugs [51, 86, 93] and maintain good hydration when the child has a fever [94].
		Hypothermia Treatment	**NEONATES** • After delivery, babies with COVID-19 are kept dry, given stimulation, warmed by the mother and breastfed immediately [57]. However, bathing is not recommended to prevent hypothermia and the spread of nosocomial infections [56].
	Tissue Perfusion	Fluid Management	• Adjust the patient’s fluid volume according to hemodynamic conditions. The amount of fluid restriction is adjusted according to the Holliday-Fresh formula [78], then record the hourly fluid intake, and output [25]. • Provide supportive care by providing, and maintaining a balanced body fluid intake, and adequate calories [14, 80, 89, 95] • Assess fluid status, and heart function before starting MISC (multisystem inflammatory syndrome-Children) treatment in children with COVID-19 [96].

COVID-19: Coronavirus Disease-2019, ETT: Endotracheal Tube, FiO_2_: Fraction of Inspired Oxygen, H_2_O: Dihydrogen Monoxide, HEPA: High-Efficiency Particulate Air, LBW: Low Birth Weight, NETS: Newborn Emergency Transport Service, NIV: Non-invasive Ventilation, OI: Oxygenation Index, OSI: Oxygen Saturation Index, PARDS: Pediatric Acute Respiratory Syndrome, PaO_2_: Partial Pressure of Oxygen, PICU: Pediatric Intensive Care Unit, PPE: Personal Protective Equipment, SpO_2_: Peripheral Capillary Oxygen Saturation.

The number in the point of intervention column corresponds to the order of the articles in S3 Table.

As shown in Table 2, the domain with the highest number of intervention recommendations was the Safety domain in the Risk Management class, which described the intervention recommendations for Infection Control (61 interventions) and Vital Sign Monitoring (six interventions). The domain with the lowest number of intervention recommendations was the Behavioral domain, which consisted of the Behavioral Therapy and Coping Assistance classes. A total of seven recommendations for Therapeutic Play and Counseling interventions were drawn from those two classes (see Table 2).

**Table 2:** Behavioral and safety domain.

Domain	Class	Intervention	Summary of Intervention
(3) Behavioral	Behavioural Therapy	Therapeutic Play	• Develop children’s coping mechanisms through play activities according to their development [70]. • Intervene based on age to address and prevent psychological problems in children during the COVID-19 pandemic **such as:** • Singing or music therapy, making music with a bowl and spoon [31, 97, 98]. • Reading books and drawing [98]. • Helping school lessons, talking about what children like [98]. • Sports, or doing tasks together [98]. • Playing collaborative games [31]. • Painting, or playing with children [69]. • Playing the peek "a-boo" [99]. • Meet the needs of communication and emotional support with children and families [101]. • Offer mental health services to children, adolescents, and families by telephone or face-to-face [102].
	Coping Assistance	Counselling	• Facilitate all questions and concerns of patients, parents or other families with open communication, understanding and patience [103]. • Monitor and identify changes in mental health status, coping, and causes of pandemic stress in children and families, overcome if problems are identified [102, 104]. • Identify the needs of mental health in children with mental health and behavioural disorders [105]. • Provide palliative care based on the basic principles of palliative care for children [106]. • Avoid separating children from family, ask children to talk about their feelings [69].
(4) Safety	Risk Management	Infection Control	• Triage nurses must use a medical mask then sort patients based on their risk level for COVID-19 infection [50, 107]. • Perform rapid screening of pediatric patients with suspected and confirmed COVID-19 [108]. Modify the triage flow arrangement and management of patient admissions to the hospital. separating patients who are at risk and not are essential [109–111]. • Health workers must wear PPE /special PPE for COVID-19 when performing procedures: delivery of infected or suspected COVID-19 mothers, preparation and administration of chemotherapy, triage, administration of inhalers and nebulizers, collection of respiratory diagnostic specimens and resuscitation [50, 57, 103, 112, 113]. Follow the general principles of management of patients who are at high risk of producing aerosol or AGP (aerosol-generating procedure) [114, 115]. • Management of PPE to prevent shortages [116]. • Wearing powered air-purifying respirators and also need to limit the number of health workers in the room [52, 57, 71, 86, 117–120]. Provide training related to the use of PPE for health workers [121]. • Invasive airway examinations or procedures and examination of the mouth cavity should not be performed if appropriate PPE is not used [50]. • Keep using a surgical mask when handling non-COVID-19 pediatric patients [50]. • Recommend that asymptomatic patients wear medical masks (especially N95) or respirators (especially FFP3), observe provisions for mask disposal [122]. • A HEPA (high-efficiency particulate air) filter can be attached to a BVM (bag valve mask), mechanical ventilator and non-invasive ventilation or aerosol box placement during intubation and suction procedures to reduce the spread of the virus [56, 74, 120, 123, 124]. • Introduce masks to children, explain the benefits of masks, and help children learn to understand the expressions and feelings of the eyes and eyebrows [99, 125–127]. • Perform nasopharyngeal/oropharyngeal swabs in a child-friendly environment and explain the test procedure to the older child [107]. • Use a single room with negative pressure for pediatric patients with suspected/confirmed COVID-19 [25, 70, 71, 86, 117, 128]. • Place contaminated clothing and sheets in infectious trash bags [106, 119]. • Early identification of infected children for immediate isolation if infected because children can become seriously ill after infection [129]. • Discontinue isolation after two negative laboratory results or with a symptom-based strategy [86, 95]. • Daily disinfection of all equipment and surfaces in the isolation room. Put trash from the isolation room separately from trash from other rooms [25, 130]. • Physical isolation for children who will undergo cancer treatment and kidney donors/recipients who will undergo transplantation surgery [87, 119, 131]. • Teach children about how to protect themselves from COVID-19 during clinic visits and evaluate knowledge about efforts to reduce the risk of infection in children [65, 104, 113, 132]. • Limit the number of visitors or people accompanying children who are hospitalized (1 member of family/ child) [87, 104, 109, 113, 116]. Perform temperature checks on all visitors before entering the hospital or NICU room [67]. • Maintain and increase awareness of maintaining hand hygiene, and physical-distancing. Use PPE (especially face masks) to reduce the spread of infection [111, 133]. • Consider the use of oxygen because it is potentially dangerous if it is not controlled [83]. • Put on a medical mask on children who use nasal prongs [86]. • Supervise the use of masks in children by parents [134]. • Protect healthcare workers and pediatric surgical patients from contracting COVID-19 [135]. • Cleaning the operating room and instruments using a disinfectant solution or UV light [118]. • Postoperative patient follow-up virtually whenever possible [136]. **NEONATES** • Prevent the spread of infection rooming-in mother-infant [55–59, 62]. • Maintain hand hygiene by washing with soap and water or alcohol before and after breastfeeding and touching babies or expressing breast milk [67]. • Routinely disinfect surfaces, baby drinking utensils and breast pumps [59, 66, 67]. • Mothers should wear surgical masks when breastfeeding babies and in close contact with babies [55–59, 62]. • The mother-infant room should be isolated from visitors [55–59, 62]. • Place the baby at a safe distance (2 meters) [55–59, 62]. • Avoid using disinfectants in breast milk containers [137]. • Disinfection of care areas for neonates with suspected/confirmed COVID-19 using 0.5% sodium hypochlorite, 70% alcohol, and hydrogen peroxide [56, 138]. • Do not delay clamping of the umbilical cord [56, 138–140]. • Avoid skin-to-skin contact of newborns with COVID-19 mothers [56, 138–142]. • Isolate and use a negative pressure incubator on babies who are at risk of COVID-19 [60, 67, 111, 143, 144]. • Advice parents to use surgical masks and wash hands before entering the NICU room [64, 67, 144]. • Isolate separately the baby who is positive for COVID-19 for 14 days [55, 69, 111, 120, 138, 140]. • Use PPE (gown, gloves, eye protection, N95 mask) when caring for premature babies suspected of having COVID-19 [88, 138]. • Do not use surgical loupe as a substitute for protective eyewear [88]. • Use P2 / N95 respirator (valveless), smoke evaluation kit, eye protection, powered air-purifying respirators (PARS) in aerosol-generating procedures [55, 67, 88, 143]. • Infant resuscitation is carried out by experienced health professionals and minimizes the number of health worker [74]. • Immediately bathe the baby after birth [145]. • Limit the number of visits to the neonatal unit [60, 64, 67, 133, 146]. • Immediately carry out the discharge of post-partum mothers [99] • COVID-19 examinations in infants with positive mothers for COVID-19 [60, 61]. • Monitor for signs and symptoms of infection in neonates [60]. Give a minimum distance of 6 feet to babies who have recently been positive but did not get AGP (aerosol-generating procedures) [67]. • Rooming-in is carried out if the mother has two negative nasopharyngeal swabs and the clinical condition improves [61]. • If rooming-in, teach parents to use masks and wash their hands regularly [68, 147]. • Use a face mask when breastfeeding as a hygiene measure [143]. • Infants infected with COVID-19 or at risk of contracting COVID-19 must be outpatient 14 days after discharge from the hospital [61]. Disinfectant: • Disinfection of the room per shift after mother and baby are discharged [64]. • Disinfect inanimate objects using diluted bleach [148]. • Use a disinfectant containing 62–71% ethanol [148]. • Perform UV irradiation for 24 hours and hydrogen peroxide in the operating room that COVID-19 patients have used [148]. • Use povidone-iodine or chlorhexidine on open wounds that are at risk of contracting COVID-19 [148]. • Disinfect large surfaces with 0.5% sodium hypochlorite (5000 ppm) and small appliances with 70% ethyl alcohol [73]. • Thoroughly clean the instrument used to measure the baby’s vital sign [140].
		Vital Sign Monitoring	• Monitor for signs and symptoms of infection in neonates [60] and children with a picture of pneumonia related to the need for ICU care [149]. • Measure vital sign before receiving (intravenous immunoglobulin) in patients with COVID-19 children with MIS_C (Multisystem inflammatory syndrome in children) as a vital sign baseline [96]. • Regular vital sign monitoring after administration of IVIG (intravenous immunoglobulin) [96]. • Ensure the patient’s blood pressure is stable before getting IVIG (intravenous immunoglobulin) [96]. • Monitoring and documenting vital sign regularly, especially SpO_2_, RR and HR [86]. • Closely monitor any worsening of symptoms (such as tachypnea, respiratory distress, or dehydration) and outpatient follow-up (children with illness) suspected or confirmed COVID-19 [104].

COVID-19: Coronavirus Disease-2019, FFP: Filtering Face Piece, HR: Heart Rate, ICU: Intensive Care Unit, NICU: Neonatal Intensive Care Unit, PPE: Personal Protective Equipment, RR: Rate Respiration, SpO_2_: Peripheral Capillary Oxygen Saturation, UV: Ultraviolet.

The number in the point of intervention column corresponds to the order of the articles in S3 Table.

Table 3 shows the Family domain, which consisted of two intervention themes: Family Presence Facilitation and Family Support. Twenty-seven recommendations were identified in the intervention themes. Furthermore, the Health System Mediation class in the Health System domain provided 33 recommendations for Discharge Planning interventions.

**Table 3:** Family and health system domain.

Domain	Class	Intervention	Summary of Intervention
(5) Family	Lifespan Care	Family Presence Facilitation	• Parents can accompany the child to use their mask, wash their hands and maintain normal temperature [68, 150]. • Facilitate the presence and communication of parents with children in the PICU room [25]. • Involve other family members to increase family cohesion and support [151]. • Facilitate parents to contact health service through audio visual services [68]. **NEONATES:** • Only parents or primary supporters are allowed to visit the NICU indefinitely [144]. • Promote skin-to-skin contact without time limit and safe breastfeeding for babies who are not isolated [144, 152]. If the mother is quarantined, discuss family members who will do skin-to-skin contact [144]. • Maximize interaction with babies whether using / not wearing masks or using transparent masks as an alternative to connecting and communicating with babies [99, 125–127]. • Observe for excessive stress in infants who are separated from the mother and make sure the baby gets a touch of caregivers [152]. • Observe the symptoms of stress, anxiety and depression in the parents due to separation and restrictions with the baby [60]. • Provide support and facilitate communication when the mother-baby condition is separated [138].
		Family Support	Family-Centered Approach [153, 154]: • Joint decision making: Facilitate 2-way communication so that parents feel calmer [63, 153, 154]. Separating babies and mothers who are positive for COVID-19 must follow the existing provisions [74]. • Parental ties: • Help the family manage stress due to separation of children and families during the treatment period [70, 153, 154] • Provide support for parents via telephone or social media [70, 119]. • Intensively inform and motivate parents to give love to children with cancer during isolation [155]. • Facilitate contact between parents and children through audio-visual [68, 153, 154]. • Avoid separating healthy babies from mothers with suspected/confirmed COVID-19 with a health condition that does not require treatment [144, 152]. Unless, temporarily separate mother and baby if the mother is symptomatic of respiratory tract infections until the test result is negative [59, 152]. • Provide kangaroo care when a positive COVID-19 mother visits the NICU [67]. Communication • Convey the child’s condition with age-appropriate communication techniques (playing or telling stories) to children who are already able to understand [79, 150]. • Provide information and emotional support on the reasons for isolation and the risks if not isolation [25, 153, 154]. • Inform and actively involve parents regarding illness and child care plans [25, 58, 91, 142, 147, 156]. • Encourage parents to breastfeed according to scientific evidence [68]. • Collaboration between disciplines to provide support for parents [68, 70]. Transparency • Openly explain to the family the reasons for limiting visits [91]. • Limit one person to watch except for the end-of-life conditions [119]. • Inform the guidelines that apply to health services related to COVID-19 [67, 153, 154]. • Care with socio-culture • Provide culturally and linguistically sensitive nursing care [153, 154]. Give a touch to the baby experiencing stress due to separation from the mother [152].
(6) Health System	Health System Mediation	Discharge Planning	• Educate what to do at home and make sure the patient and family are ready to carry out medical procedures after being discharged [64, 142, 156, 157]. • Education for self-isolation at home for 14 days after treatment [60, 64, 86, 92, 142, 156, 158]. • In children with nephrotic syndrome, give education related to management, clinical status and home care plans [104] • Children are allowed to go home if the inflammation values are typical, no fever, normotensive, good hydration, and without oxygen support [159]. • Discharge planning care in the mother’s room or via video or telephone lines [124, 160]. • Provide educational media such as leaflets [64]. • Practices appear to have the capacity to deliver routinely recommended vaccines, allowing children who have missed vaccine doses because of the pandemic to catch up. Practices that are unable to provide immunization services should refer patients to other practices [75]. • Utilizing telemedicine/telehealth for follow-up and meeting patient needs [61, 64, 65, 87, 90, 92, 113, 146, 161–167]. **NEONATES** • If the baby is not rooming-in to the mother, she/he can be discharged from the hospital at the age of 24–48 hours [56]. • Before going home, educate parents about signs and symptoms of danger to neonates [56, 64]. • If the parents are positive, suggest care-giver from family member who has been COVID-19 negatively tested to care for the newborn in the home [124]. • Suggest doing basic vaccinations on schedule [64]. • Teach the mother to keep a distance and use a mask if there are still symptoms until seven days after the symptoms appear [61].
		Case Management	• Advise parents to communicate with children via video [168]. • Physical examination is carried out only on the part related to complaints [92]. • Non-chemotherapy transfusions and infusions in children with cancer can be given at home [103]. • Answer children’s questions honestly regarding COVID-19 according to their stage of development [65, 70]. • Facilitate children in expressing their needs [70]. • Create a protocol that allows the patient’s environment to remain safe during assessment and specimen collection [70, 91, 165]. • Ensure that neonates will be handled by well-trained health care personal [91, 119]. • Collaboration with parents regarding IEP (Individualized Education Program) in children with disabilities [169]. • Traffic restrictions among COVID-19 patients [140]. • Inform about the impact of COVID-19 on families [146]. • Communicate regarding the patient’s needs, discomfort, and support for the patient during the treatment process [129]. • Meet regularly (2 times a week) at the pediatric department to discuss developments related to COVID-19 management [116]. • Interdisciplinary collaboration and involving stakeholders are needed if urgent elective surgery is performed [170]. Actively communicate the surgical plan and reasons for postponing surgery to parents regarding COVID-19 [170]. • Monitoring children’s habits related to screen-time, sleep patterns, physical activity, habits, eating, and psychological responses [171]. • Carry out family tracing and activities on children infected with COVID-19 [172]. **NEONATES:** • Follow the modified algorithm directions by the neonatology team in case of the emergency airway in neonates [154]. • Modification of postnatal management of neonates from COVID-19 positive mothers if a separate isolation room is available or not available at the hospital [73]. • Do a neonatal COVID-19 test within 24 hours of birth to avoid false positives [67, 123]. • Provide supportive care to newborn infected with COVID-19 [138].

COVID-19: Coronavirus Disease-2019, NICU: Neonatal Intensive Care Unit, PICU: Pediatric Intensive Care Unit.

The number in the point of intervention column corresponds to the order of the articles in S3 Table.

## Discussion

This scoping review was conducted to provide nursing intervention recommendations for nursing staff caring for pediatric patients with COVID-19 in the hospital setting. To the best of our knowledge, this study is the first to review the scope of the literature on the comprehensive nursing care of pediatric patients with COVID-19. Previous systematic and scoping reviews concerned epidemiological studies [23, 173], nursing approach in diagnosing COVID-19 in children [174], and patients’ clinical manifestations [13, 20]. Other previous studies discussed nurses’ efforts in preventing and controlling the spread of COVID-19 infection in neonates [175], improving clinical service in the maternity field [176], newborn care during the COVID-19 pandemic [177], and palliative care for people with dementia [178]. However, comprehensive reviews of nursing care in pediatric patients with COVID-19 are limited. Our research was conducted using a systematic method with a rigorous approach, which should be considered credible.

The three biggest domains revealed in this study are Safety, Health System, and Physiological: Basic which are comprised 79, 44, and 37 point of nursing interventions, respectively. Thus, Infection Control, Positioning, and Discharge Planning will be discussed as representative from those domains. Furthermore, we also highlight Ventilation Management: Invasive, Hyperthermia Treatment, Discharge Planning, Family Presence Facilitation, and Therapeutic Play because of their potency to fulfill practice gap in pediatric nursing alongside COVID-19 context.

### Infection control

Infection control strategies are the main points highlighted in preventing the transmission of COVID-19 or SARS-CoV-2 infection. Nurses play an important role in infection prevention and control through some measures in daily practices [174]. Various changes in interventions occurred in the care of pediatric patients during the pandemic, but the emphasis of the recommendations that have been made still on the use of Personal Protective Equipment (PPE). Health workers must use PPE properly to protect themselves and prevent cross-infection, so training on the use of PPE needs to be provided for health workers [121]. In addition, nurses also need to follow the general principles of managing patients undergoing aerosol-generating procedures (AGP) [114, 115] when performing AGP such as chest compressions, airway management, ventilation, and suction [74] who are at high risk of transmitting infection. The use of PPE must be rational according to the setting, personnel, and type of activity [179].

Neonatal care settings are undergoing substantial changes. Before COVID-19, the WHO recommendation on the timing of the newborn’s first bath should be postponed until 24 hours after birth [180] considering the incidence of hypothermia in newborns. The results of a recent study even suggested delaying the first bath up to 48 hours after delivery to effectively maintain the baby’s body temperature and effectively maintain skin moisture which can have a positive impact on the development of the baby’s skin [181]. Unlike during COVID-19, babies are bathed immediately after birth to clean viral particles obtained from environmental exposure [145].

The direct contact of the baby with the mother, which was previously the main intervention carried out after birth, has turned into something that is not done or postponed until it meets the safety criteria from the transmission of COVID-19 infection. This context is the highlight of this scoping review. A total of 21 papers describe the regulation of contact between infants and mothers who are infected with COVID-19 or at risk of COVID-19. The types of interventions recommended by previous authors include avoiding skin-to-skin contact [56, 138–142], isolation of infants in negative pressure incubators [60, 67, 111, 143, 144] or isolation separately from the mother for 14 days [55, 77, 111, 120, 138, 140], rooming-in provision [61, 68, 147], and wear a face mask when breastfeeding or in close contact with infants [55–59, 62, 143].

### Positioning

The prone position is highly recommended for COVID-19 patients with acute respiratory distress syndrome (ARDS) [182]. Early prone positioning is recommended for pediatric patients with moderate to severe pediatric acute respiratory distress syndrome (PARDS) for 12–18 hours per day (avoid disconnection) to improve the oxygen state [79]. The average time of prone positioning in children and adults is similar. In adult COVID-19 patients with severe ARDS, prone position can increase PaO2:FiO2, primarily in patients with PaO2:FiO2 <120 mm Hg. It can be delivered five sessions per day was 14 hours [183]. Another study noted that after 16 hours of prone position per day in adult COVID-19 patients, moderate to severe ARDS reduced mortality and improved physiological parameters [184]. The respiratory system mechanics of patients with ARDS, with or without COVID-19, were broadly similar, and the management of ARDS patients with or without COVID-19 was similar [185]. Multifactorial factors plays a role in improving oxygenation during prone ventilation by reducing lung compression and improving lung perfusion and changes in the distribution of extravascular lung fluid and secretions [186].

### Discharge planning

Discharge from hospital to home or to another level of care is a transition situation in pediatric care [47, 187]. Nurses’ activities in managing patient discharge include providing patient care knowledge and skills, patient teaching, and post-discharge evaluation [47]. Discharge planning intervention recommendations were found to differ in knowledge preparation, re-examination, and post-treatment care. Before discharging a pediatric patient with COVID-19, children and families should be provided with knowledge about the post-treatment isolation period [60, 64, 86, 92, 142, 156, 158] and family tracing [172]. In addition, it must be ensured that newborns are cared for by parents or caregivers who are free from COVID-19 infection [124]. It is also important for nurses to educate patients and families about recognizing the signs and symptoms of danger to neonates and ensure that they have the ability to perform post-discharge medical procedures [68, 76, 119, 153]. The spread of infection from patients and families must be controlled by nurses by providing appropriate discharge education to prevent the spread of COVID-19.

Use of technology as telemedicine/telehealth for follow-up and fulfillment of patient needs [61, 64, 65, 87, 90, 92, 113, 146, 161–168] after discharge, seems to be the main issue in providing child services that can reduce physical contact and maintain distance during the period of social isolation or quarantine. Similar to the management of COVID-19 cases in children, the application of a safe distance in the clinical service setting [168] can limit direct contact with health workers. In this case, teleconsultation services and parental education through video media have a crucial role in maintaining the best possible service delivery to pediatric patients and their families [168].

### Mechanical ventilation management

Intubation procedures occurred in 4.5% of pediatric patients admitted to intensive care units [188]. To prevent complications and air leaks in this study, nurses should check the endotracheal tube (ETT) cuff pressure at a safe limit of <20 cm H_2_O every 6–12 hours [79]. Our findings were similar to previous studies. Kumar et al. and Bulamba et al. [189, 190] proposed a pressure target of an ETT cuff at 20–30 cm H_2_O. The correct endotracheal cuff pressure must be less than the capillary perfusion pressure or less than 30 cm H_2_O [191]. Talekar et al. [192] also suggested ETT cuff daily monitoring every 6–12 hours. Increased pressure has the potential to decrease mucosal blood flow, possibly increasing the risk of subsequent tracheal stenosis, rupture, ventilator-associated pneumonia (VAP), and other complications [193]. Poor cuff pressure management could increase the number of days in the Intensive Care Unit (ICU) and prolong the use of mechanical ventilation [194].

### Hyperthermia treatment

Another substantial change found in our review is the attitude toward fever in pediatric patients with COVID-19. Increases in body temperature need to be closely monitored even when they are minimal [92]. A previous study found maximum body temperature among the admission factors that led to the progression of COVID-19 [195]. Moreover, severe hyperthermia was expected, and increasing temperatures were independently associated with increased mortality rates [196]. A significant increase in mortality was recorded for every 0.5°C increase in maximum body temperature during COVID-19 [197].

### Family presence facilitation

Patient and family-centered care (PFCC) has been an essential part of pediatric care during the pandemic [198]. Based on our findings, we recommend the presence of the families of pediatric patients in isolation. This recommendation is in line with former studies outside the COVID-19 context [199]. Additionally, our findings emphasized the challenges in involving families in child care with provisions for isolation and restrictions on admission to the care unit to stay safe and prevent the spread of the virus [25, 68]. Parents and caregivers must wear a mask, wash hands, and show average BT to reduce the risk of COVID-19 while accompanying the child [68, 150]. Other modes of family presence, such as virtual presence (video conference), should be considered when physical attendance is not possible [200]. However, in this mode, there are issues related to patient privacy and the exacerbation of racial, socioeconomic, and geographic disparities in populations that lack reliable internet access, devices, or technological literacy [153]. Several measures have been developed to maintain the family’s presence despite burden issues in pediatric isolation settings [153, 201].

### Therapeutic play

The COVID-19 disease causes physical and psychological problems similar to those in the Ebola epidemic [202]. Signs and symptoms of changes in the psychological health status, coping mechanism, and causes or risk factors of pandemic stress in children and their families must be identified by pediatric nurses in treating pediatric COVID-19 patients [102, 104]. Pediatric COVID-19 patients in quarantine could experience mental health problems due to the loss or separation from parents or families who suffer from chronic illnesses [32, 34]. They could also suffer stigmatization, social exclusion [35], uncertainty about disease status, boredom, and restrictions on movements and daily activity [36]. The manifestations of psychological stress are anxiety, social interaction disorders, and negative changes in children’s behavior, such as aggression and affect [151], decreased appetite, depression, lethargy, irritability, and fear [31, 32]. Psychological interventions must be provided by child health services [34, 100] to reduce stress and increase the adaptability of children and families to life during the pandemic.

The results of this review showed that various kinds of therapeutic play interventions were recommended by previous authors to address the psychological problems of children of all ages due to the pandemic, such as playing collaborative games and painting [31, 69, 100]. In pediatric patients, playing can act as a medium to reduce loneliness, provide distractions [31], and improve health status and wellbeing [203]. However, strategies for fulfilling children’s play needs during isolation or quarantine different from those before the pandemic [203]. One guideline recommended the application of safe physical distancing in children’s physical activities and playtime [204].

The findings of this scoping review indicated that the amount of literature on physical, psychological, and social care is increasing. However, studies are lacking on the spiritual care of children and preparation for the death or loss of a child, parent, or family member, which could occur suddenly. Furthermore, cultural aspects have not been considered by many previous authors. The COVID-19 pandemic has led to many cultural changes. For example, the culture of maintaining health during COVID-19 requires wearing masks, frequent handwashing, and the increased consumption of fruits and vegetables, which may present special challenges in the pediatric population. Atout et al. [151] showed that many children refused to use masks and other personal protective equipment for non-essential reasons, such as feeling uncomfortable and feeling different from their peers. Therefore, further studies should be conducted to determine cultural influences on providing holistic care in children.

### Limitations

This brief scoping review has several limitations. First, we included only articles that were published in the English language. Second, because of the heterogeneity of the design of this scoping review study, we did not perform a structured quality assessment. However, this is in line with accepted methodology of scoping review proposed by Arksey and O’Malley [43]. We accept low levels of evidence in order to understand the whole landscape of published literature.

### Implications for practice

Besides physical complaint, the COVID-19 situation has led to psychological problems in children who suffer parental separation, social isolation, and stigmatization. Pediatric nurses are expected to be able to propose various creative ways to meet the needs of children and families despite the various limitations in isolation rooms. Pediatric should promote family presence where it is possible [200]. Using technology might become a good alternative to connect children with their families where physical contact is unaffordable. Some gadgets have developed specific software that enables the child, family, and health care team to stay connected all day. Pediatric nurses should manage the daily schedule where family can connect to health care team among the other routine tasks along the shift period [198].

Furthermore, the pandemic has caused a new situation that affects the readiness of both children and families in undergoing the transition from hospitalization to isolation at home. On this occasion, discharge planning can play an essential role in preparing child and family knowledge, and skill. Parents and child should be educated about the post-treatment isolation period including routine care, nutritional and emotional support, home isolation procedures, and sign of emergency. Pediatric nurses should also help family to decide on child-caregiver at home which may lead to family dilemma. The pandemic situation has prompted pediatric nurses to show leadership in managing resources to ensure that good nursing care has been delivered to consider all needs of pediatric patients and their families.

## Conclusion

We found Safety, Health System, and Physiological: Basic as the three most domain revealed in this brief summarizing study. Accordingly, our guideline in Infection Control, Positioning, and Discharge Planning should be considered to be delivered to in-hospital pediatric nursing practice. Ventilation Management: Invasive, Hyperthermia Treatment intervention was formulated to address one of the major physical conditions in pediatric with COVID-19. Nursing intervention should pay attention to pediatric patients with psychological problem by providing Family Presence Facilitation, and Therapeutic Play. Using technology to promote proper discharge planning in pandemic context found to be essential to delivered nursing practice comprehensively. Pediatric nurses should collaborate with their interprofessional team members and use the resources available to ensure the best care despite various restrictions during the COVID-19 pandemic.

## Supporting information

S1 Checklist PRISMA-ScR checklist. (DOCX)

S1 Table Search strategy examples. (DOCX)

S2 Table Inclusion and exclusion criteria. (DOCX)

S3 Table Characteristics of included studies. (DOCX)
