# Supplementary material for: Nursing care recommendation for pediatric COVID-19 patients in the hospital setting: A brief scoping review
Source: PLoS One. 2022 Feb 3;17(2):e0263267. doi: 10.1371/journal.pone.0263267 (PMC8812980; doi:10.1371/journal.pone.0263267)
Supplement: S1 Table — (DOCX) [file pone.0263267.s002.docx]

**S1 Table. Search strategy examples**

| **CINAHL (n=509)** |
| --- |
| =(+Pediatric+OR+Pediatrics+OR+Child+OR+Children+OR+newborn+OR+neonates+)+AND+(+Nursing+Care+OR+Nursing+intervention+Or+nursing+consideration+OR+Nursing+Guidance+OR+Nursing+Management+OR+Nursing+Protocol+OR+Nursing+Treatment+OR+Nursing+Recommendation+)+AND+(Covid-19+Corona+Coronavirus+2019-nCoV+SARS-CoV) |
| **Science Direct (n = 909)** |
| (Pediatric OR newborn) AND (nursing management OR nursing intervention OR protocol)) AND (covid-19 OR coronavirus))) |
| **Proquest (n = 105)** |
| (Pediatric OR Pediatrics OR Child OR Children OR newborn OR neonates) AND (Nursing Care OR Nursing intervention Or nursing consideration OR Nursing Guidance OR Nursing Management OR Nursing Protocol OR Nursing Treatment OR Nursing Recommendation) AND (Covid-19; Corona; Coronavirus; 2019-nCoV; SARS-CoV-2) |
| **Embase (n = 23)** |
| (('pediatric'/exp OR pediatric OR 'pediatrics'/exp OR pediatrics OR 'child'/exp OR child OR 'children'/exp OR children OR 'newborn'/exp OR newborn OR neonates) AND ('management'/exp OR management) OR 'protocol'/exp OR protocol OR 'treatment'/exp OR treatment OR recommendation OR 'intervention'/exp OR intervention OR 'nursing care'/exp OR 'nursing care' OR (('nursing'/exp OR nursing) AND ('care'/exp OR care)) OR 'nursing intervention'/exp OR 'nursing intervention' OR (('nursing'/exp OR nursing) AND ('intervention'/exp OR intervention)) OR 'nursing consideration' OR (('nursing'/exp OR nursing) AND consideration) OR 'nursing guidance' OR (('nursing'/exp OR nursing) AND ('guidance'/exp OR guidance)) OR 'nursing management'/exp OR 'nursing management' OR (('nursing'/exp OR nursing) AND ('management'/exp OR management)) OR 'nursing protocol'/exp OR 'nursing protocol' OR (('nursing'/exp OR nursing) AND ('protocol'/exp OR protocol)) OR 'nursing treatment' OR (('nursing'/exp OR nursing) AND ('treatment'/exp OR treatment)) OR 'nursing recommendation' OR (('nursing'/exp OR nursing) AND recommendation)) AND ('covid-19; corona; coronavirus; 2019-ncov; sars-cov-2' OR ('covid 19;' AND corona; AND coronavirus; AND '2019 ncov;' AND ('sars cov 2'/exp OR 'sars cov 2'))) |
| **SpringerLink (n = 14)** |
| "Pediatric OR Pediatrics OR Child OR Children OR newborn OR neonates" anywhere and "Nursing Care OR Nursing intervention Or nursing consideration OR Nursing Guidance OR Nursing Management OR Nursing Protocol OR Nursing Treatment OR Nursing Recommendation" anywhere and "Covid-19; Corona; Coronavirus; 2019-nCoV; SARS-CoV-2" anywhere |
| **Taylor & Francis (n = 15)** |
| [[All: pediatric] OR [All: pediatrics] OR [All: child] OR [All: children] OR [All: newborn] OR [All: neonates]] AND [All: nursing] AND [[All: care] OR [All: nursing]] AND [All: intervention or nursing] AND [[All: consideration] OR [All: nursing]] AND [[All: guidance] OR [All: nursing]] AND [[All: management] OR [All: nursing]] AND [[All: protocol] OR [All: nursing]] AND [[All: treatment] OR [All: nursing]] AND [All: recommendation] AND [All: covid-19; corona; coronavirus; 2019-ncov; sars-cov-2] AND [Publication Date: Last Year] |
