# Supplementary material for: Nursing care recommendation for pediatric COVID-19 patients in the hospital setting: A brief scoping review
Source: PLoS One. 2022 Feb 3;17(2):e0263267. doi: 10.1371/journal.pone.0263267 (PMC8812980; doi:10.1371/journal.pone.0263267)
Supplement: S2 Table — (DOCX) [file pone.0263267.s003.docx]

**S2 Table. Inclusion and exclusion criteria**

| **Inclusion** | **Exclusion** |
| --- | --- |
| Children ages range 0-19 years |  |
| Intervention/protocol/guidelines/clinical recommendation | Systematic review, scoping review article, & Meta-analysis |
| Report the clinical recommendation in delivering nursing care for paediatric with COVID-19 |  |
| Peer-reviewed articles using observational or interventional study, or |  |
| Grey literature including policy papers, guides or guidelines, letters and editorials, newspaper or web articles |  |
| Published with the period of December 2019 up to 24^th^ January 2021 |  |
