# Supplementary material for: Nursing care recommendation for pediatric COVID-19 patients in the hospital setting: A brief scoping review
Source: PLoS One. 2022 Feb 3;17(2):e0263267. doi: 10.1371/journal.pone.0263267 (PMC8812980; doi:10.1371/journal.pone.0263267)
Supplement: S3 Table — (DOCX) [file pone.0263267.s004.docx]

**S3 Table. Characteristics of included studies**

| **Number** | **First author** | **Country** | **Type of article** | **Purpose of the article** |
| --- | --- | --- | --- | --- |
| (1) | Abuali et al.  (2021) | USA | Review article | To develop innovation protocols at an Academic Paediatric practice during the COVID-19 (2019 novel corona-virus) crisis |
| (2) | Ahmad et al.  (2020) | Saudi Arabia | Review article | To explain new perspective approach to explore the pros and cons of face masks in preventing the spread of SARS-CoV-2 and other pathogens |
| (3) | Al Juhani et al.  (2020) | Saudi Arabia | Review article | To describe preparation for the management of children who are confirmed or suspected in the hospital perioperative period |
| (4) | American Academy of Pediatrics  (2020) | USA | Clinical guidance | To provide recommendation management of infants born to mothers with suspected or confirmed COVID-19 |
| (5) | Ashokka et al.  (2020) | Singapore | Clinical opinion | To describe management strategies derived from best available evidence to provide guidance in caring for the high-risk and acutely ill parturient |
| (6) | Atout et al.  (2021) | Jordania | Research article | To investigate the experiences of parents who care for children diagnosed with leukaemia in the context of the COVID-19. |
| (7) | Aubey et al.  (2020) | USA | Review article | To describe inpatient management strategies and considerations for pregnant patients with severe acute respiratory syndrome coronavirus 2 infection |
| (8) | Barney et al.  (2020) | USA | Review article | To describes the rapid implementation of telemedicine within an adolescent and young adult (AYA) medicine clinic in response to the Coronavirus Disease 2019 (COVID-19) pandemic |
| (9) | Udea et al.  (2020) | United States | Case Report | To describe severe COVID cases in pediatric patient |
| (10) | Boelig et al.  (2020) | USA | Review article | To provide guidance regarding methods to appropriately screen and test pregnant patients for COVID-19 |
| (11) | Bogiatzopoulou et al.  (2020) | UK | Review article | To detect early and late manifestations of the disease. |
| (12) | Bouffet et al.  (2020) | UK | Commentary | To provide advice and management of children with cancer during the COVID-19 pandemic |
| (13) | Bressan et al.  (2020) | Europe | Review article | To describe the variability and identify gaps in preparedness and response to the COVID-19 pandemic in European EDs caring for children |
| (14) | Campagnaro et al.  (2020) | Brazil | Research article | To assess the impact of the pandemic on fear, dietary choices and oral health perceptions of parents. |
| (15) | Carlotti et al.  (2020) | Brazil | Review article | To verify the main epidemiologic, clinical, laboratory-related, and therapeutic aspects of coronavirus disease 2019 in critically ill paediatric patients |
| (16) | Castillero et al.  (2020) | USA | Clinical guide | To provide a comprehensive overview of neonatal–perinatal perspectives of COVID-19, ranging from the basic science of infection and recommendations for care of pregnant women and neonates to important psychosocial, ethical, and racial/ethnic topics emerging as a result of both the pandemic and the response of the healthcare community to the care of infected individuals. |
| (17) | Cavicchiolo et al.  (2020) | Italy | Review article | To identify safe standardized operational procedures involving the hospitals of the network area, the personnel involved in NETS, and the hub centres. (Fig. 1) Two dedicated ambulances, transport incubators, and emergency bags were exclusively deputed to the transport of at-risk neonates. |
| (18) | Center for Disease Control and Prevention  (2020) | USA | Guide | To describe strategies or options to optimize supplies of disposable N95 filtering face piece respirators |
| (19) | Chawla et al.  (2020) | India | Recommendations | To provide recommendations for prevention of transmission, diagnosis of infection and providing clinical care during labour, resuscitation and postnatal period |
| (20) | Chen et al.  (2021) | USA | Review article | To optimize the quality of perinatal care during the pandemic, appropriate mental health interventions must be implemented to prevent and alleviate perinatal anxiety and depression and improve maternal and infant outcomes |
| (21) | Chen et al.  (2020) | China | Case report | To further standardize the protocol for diagnosis and treatment of respiratory infection in children caused by 2019-nCoV |
| (22) | (Choi et al.  (2020) | USA | Recommendations | To provide recommendations for practice, research, and policy related to mental health in the perinatal period include the use of a trauma-informed framework to promote social support and infant attachment, use of technology and telehealth, and assessment for mental health needs and experiences of violence |
| (23) | DeFazio et al.  (2020) | USA | Guide | To describe guidelines for paediatric surgical decision making during the COVID-19 pandemic |
| (24) | Aleebrahim-Dehkordi et al.  (2020) | Iran | Review article | To describe current understanding of SARS-CoV-2 infection in the paediatric age group compared with MERS-CoV and SARS-CoV. |
| (25) | Deitrick et al.  (2020) | USA | Review article | To provide guidelines for treating Covid-19 patients in the emergency room |
| (26) | Deprest et al.  (2020) | USA | Original article | To review relevant recent information to optimize prenatal care delivery |
| (27) | Devrim and Bayram  (2020) | USA | Review article | To share experiences of how to handle patients with COVID-19 in a paediatric referral and tertiary care hospital to prevent the possible transmissions to the healthcare workers (HCWs) |
| (28) | Ding et al.  (2020) | China | Guidance | To provide guidance on the management of paediatric fever clinics during the CoV pneumonia epidemic period, which outlines in detail how to optimize processes, prevent cross-infection, provide health protection, and prevent disinfection of medical staff |
| (29) | Dokken et al.  (2020) | USA | Original article | To review specific programs of Children's Mercy with hospitals in both Kansas City, MO, and Overland Park, KS, as well as several other institutions, and outlines resources for nurses to share with families. |
| (30) | Dumpa et al.  (2020) | USA | A case report and review of literature | To explain case highlights the milder presentation of COVID-19 in otherwise healthy, full-term neonates. |
| (31) | ELSO  (2020) | Australia | Guideline | To assist existing extracorporeal membrane oxygenation (ECMO) centres to prepare and plan provision of ECMO during the ongoing pandemic |
| (32) | Enyama et al.  (2020) | Cameroon | Original article | To describe the impact of the COVID-19 pandemic on the clinical activity of paediatricians |
| (33) | ESPNIC  (2020) | Europe | Guide | To provide nursing guidance for the care of the child with suspected or proven COVID-19 infection |
| (34) | Evans et al.  (2020) | USA | Commentary | To identify using telemedicine to reach adolescents during the COVID-19 pandemic |
| (35) | Ezenwa et al.  (2020) | Nigeria | Guideline | To describe the guideline prepares and equips clinicians working in the maternal and new- born sections in the sub-region to manage COVID-19 during pregnancy and childbirth |
| (36) | Farshi et al.  (2020) | Iran | Case report | To provide ursing care in a child with coronavirus disease 2019 |
| (37) | Frauenfelder et al.  (2020) | UK | Guideline | To describe the guidelines for the operation of otolaryngology cases in children during the COVID-19 pandemic. |
| (38) | Gale et al.  (2021) | UK | Review article | To describe the incidence, characteristics, transmission, and outcomes of SARS-CoV-2 infection in neonates who received inpatient hospital care in the UK |
| (39) | Gerall et al.  (2020) | AS | Review article | To treat acute appendicitis in paediatric patients during the peak of the COVID-19 outbreak in New York City (NYC) and highlight the importance of providing patients with continued access to medical care during a pandemic when resources are limited and fears of infection high. |
| (40) | Giuliani et al.  (2020) | Italy | Expert opinion | To provide following suggestions on breastfeeding during the COVID-19 pandemic addressed both to mothers with and without diabetes |
| (41) | Gralton et al.  (2020) | USA | Special article | To describe the Department of Nursing Research and EBP continues to be available for consultation and mentoring of staff, as staff use current evidence to drive continued practice changes and consider new projects |
| (42) | Green et al.  (2020a) | Australia | Integrative review | To analyse literature related to neonatal nursing practice during the COVID-19 pandemic |
| (43) | Green et al. (2020b) | Australia  UK | Discussion paper | To discuss paper focuses on an exploration of these factors in the light of the potential impact of COVID-19 restrictions on neonatal care. |
| (44) | Green et al.  (2021) | Australia | Review article | To discuss the potential impact of wearing face masks in the new born period in line with underpinning theory, creating key recommendations for practice |
| (45) | Greene et al.  (2020) | USA | Retrospective cohort study | To determine whether labour and delivery unit policy modifications we made during the coronavirus disease 2019 pandemic were associated with differences in outcomes for mothers and new-borns. |
| (46) | Hahn and Vater  (2020) | USA | Web page | To describe COVID-19 and Kids: Managing Chronic Pain at Home |
| (47) | Hanekamp and Huang (2020) | USA | Web page | To provide general information about COVID-19 |
| (48) | Hart et al.  (2020) | USA | Guidance | To describe FCC during The Covid-19 era |
| (49) | Hester  (2020) | USA | Guidance | To provide the guidance issued by the American Academy of Paediatrics (AAP) for caring of new-borns who are born to mothers with COVID-19. |
| (50) | Hester  (2021) | USA | Review article | To discuss ADHD in the COVID era |
| (51) | Houtrow et al.  (2020) | USA | Position paper | To provide a discussion of the following topics: the immediate and ongoing impacts on children with disabilities and their families, the ethical concerns and implications of triage protocols for scarce resources that consider disability in their scoring systems, and optimizing medical care and educational needs in the time of COVID |
| (52) | Human Milk Banking Association of North America  (2020) | USA | Guideline | To suggest guideline milk handling for COVID-19 positive or suspected mothers in the hospital setting |
| (53) | Ingram et al.  (2020) | USA | Review article | To describe an initial evaluation of self-reported paediatric surgical policy changes from hospitals across North America |
| (54) | Jain et al.  (2020) | USA | Review article | To discuss testing and cohorting of patients, personal protective equipment utilization, limiting workplace exposure, and information sharing |
| (55) | Jiang et al.  (2020) | USA | Review article | To inform COVID-19 and multisystem inflammatory syndrome in children and adolescents |
| (56) | Jiao et al.  (2020) | Europe | Review article | To raise awareness regarding children’s psychological needs during epidemics and report early data collected in the COVID-19 affected areas in China during the current outbreak, emphasizing the role of families and caregivers in the timely recognition and management of negative emotions. |
| (57) | Johnson and Dupler  (2020) | New York | Literature article | To understanding the health conditions of children affected by delayed procedures is very important for their well-being and development |
| (58) | Joosten et al.  (2019) | Netherlands | Review article | To proposes strategies and considerations for nutritional support during the recovery phase to gain optimal (catch-up) growth with preservation of lean body mass. |
| (59) | Kache et al.  (2020) | - | Guideline/Recommendations | To provide COVID-19 PICU guidelines: for high- and limited-resource settings |
| (60) | Kallem and Sharma  (2020) | India | Expert article | To inform COVID-19 in neonates |
| (61) | Kari et al.  (2021) | Saudi Arabia | Research article | To describe characteristics of COVID-19 in children in hospitalization, clinical and risk factors for mortality, morbidity, and Pediatric Intensive Care Unit (PICU). |
| (62) | Karimi et al.  (2020) | Iran | Guide | To provide the algorithm based on the standard diagnosis and treatment strategies for pediatric viral infections and available strategies to prevention of COVID-19 infection. |
| (63) | Kassab et al.  (2020) | - | Research article | To support the use of several non-pharmacologic interventions, such as facilitated tucking by parents (FTP) for neonatal pain management |
| (64) | Kaushik et al.  (2020) | USA | Correspondence/letter of editor | To commend the authors for a timely and succinct article on approach to management of pediatric COVID-19, providing essential and practical guidance to clinicians, and would like to comment on the management of COVID-19 in light of recent evidence, available studies and guidelines. |
| (65) | Kazzaz et al.  (2020) | Saudi Arabia | Review article | To summarize important considerations for pediatric hospital preparedness at the hospital level that includes workforce, equipment, supply; capacity planning, and infection prevention strategies, it also spans over the management of COVID‑19 pediatric patients in high‑risk areas such as critical care areas, Emergency Department and operative rooms. |
| (66) | Kneyber et al.  (2020) | Europe | Consensus statement | To suggest ractice recommendations for the management of children with suspected or proven COVID-19 infections from the Paediatric Mechanical Ventilation Consensus Conference (PEMVECC) and the section Respiratory Failure from the European Society for Paediatric and Neonatal Intensive Care (ESPNIC) |
| (67) | Krishnamurthy et al.  (2020) | USA | Review article | To recognize the highly efficient transmission characteristics of SARS-C0V-2 and its potential for causing serious disease in vulnerable individuals, including health care workers |
| (68) | Kumar et al.  (2020) | India | Research article | To study Feasibility and effectiveness of teleconsultation in children with epilepsy amidst the ongoing COVID-19 pandemic in a resource-limited country. |
| (69) | Lakhani dan Sharma  (2020) | India | Recommendation and guideline | To summarize current re- commendations and our institutional guidelines for diagnosing and managing a pediatric orthopedic patient in the COVID-19 pandemic. |
| (70) | Leff et al.  (2020) | USA | Research article | To evaluate whether changes in frequency or patient demographics among children and adolescents presenting to the PED has occurred |
| (71) | López-Bueno et al.  (2021) | UK | Review article | To investigate potential health risk behaviours amongst isolated pre-school and school-aged children |
| (72) | Lopez et al.  (2020) | Salt Lake City, Utah | Research article | To inform better understand transmission from young children |
| (73) | Lotfi et al.  (2020) | Iran | Review article | To discuss current therapeutic options, preventive methods and transmission routes of COVID-19 |
| (74) | MaGowan et al.  (2020) | USA | Review article | To study a broad view of the educational needs of the clinical nurse caring for the child with multi- system inflammatory syndrome. |
| (75) | Marino et al.  (2020) | Netherlands. | Recommendation | To provide a summary of nutrition support recommendations for critically ill children with COVID-19. |
| (76) | Marraro and Spada  (2020) | Italy, China | Review  article | To provide considerations on the actual treatments, on how to avoid complications and the undesirable side effects related to them and to select and apply earlier the most appropriate treatment. |
| (77) | Matava et al.  (2020) | Canada | Guideline | To suggest the guidelines from the society for pediatric anaesthesia’s pediatric difficult intubation collaborative and the Canadian pediatric anaesthesia society |
| (78) | Michigan Department of Health and Human Services (MDHHS)  (2020) | USA | Guideline | To inform about what might happen due to the novel coronavirus (COVID-19) outbreak |
| (79) | Mihatsch et al.  (2018) | Europe | Guideline | To inform guideline development process for the updated guidelines |
| (80) | Mirlashari et al.  (2021) | Iran | Research article | To investigate the perspectives of children with cancer and their family in this era of the COVID-19 pandemic |
| (81) | Montes et al.  (2020) | Spain | Review article | To review the changes that have occurred in neonatal units, and their impact on neonatal care and families during pandemic |
| (82) | Moro and Bertino  (2020) | Italy | Review article | To inform Breastfeeding, human milk collection and containers, and human milk banking: Hot topics during the COVID-19 pandemic |
| (83) | Monzani et al.  (2020) | Italy | Research article | To document the lived experience of Italian pediatric emergency physicians during the coronavirus disease 2019 (COVID-19) pandemic |
| (84) | Mulay et al.  (2021) | Singapore | Frame work/guide | To study an adaptive framework as a large Developmental and Behavioural Pediatrics service in a tertiary academic institution in Singapore |
| (85) | Nicholas et al.  (2020) | Canada | Discussion | To describe result of roundtable discussion with pediatric specialists highlights experiences in providing care during the COVID-19 pandemic. |
| (86) | Nolan et al.  (2020) | Europe | Guideline | To give the standard resuscitation guidelines for adults and children in Europe |
| (87) | Peck  (2020) | USA | Review article | To summarize the latest evidence on the rapidly developing coronavirus pandemic |
| (88) | Peng et al.  (2020) | China | Research article | To presents the interim results of the possibility of 2019 novel coronavirus disease (COVID-19) transmission to neonates through breast milk |
| (89) | Pereira et al.  (2020) | Spain | Case study | To describe the management of the mothers and babies during breastfeeding, the indication of breastfeeding depending on the severity of symptoms and finally breastfeeding in preterm neonates during NICU admission |
| (90) | Rajapakse and Dixit  (2020) | China | Review article | To review the infections of SARS COV- 2 in children |
| (91) | Rathore et al.  (2020) | India | Case article | To discuss the concerns and reflect the issues of a 10‑year‑old boy of ALL who was tested COVID positive during the evaluation and treatment of his disease and was admitted in a COVID isolation centre along with his mother who was COVID negative. |
| (92) | Robinson et al.  (2020) | Canada | Guideline | To adapt Canadian adult glomerulonephritis guidelines to make them applicable to children and discuss pediatric specific considerations |
| (93) | Rodríguez Yago et al.  (2020) | Spain | Recommendation | To provide recommendations on the approach to cancer in patients with suspected or confirmed SARS-COV-2 infection, in any location and applicable to all healthcare professionals, based on a review of the available scientific evidence, and as an expert opinion consensus document. |
| (94) | Royal College of Paediatrics and Child Health  (2020) | UK | Consensus | To describe the current consensus on the risk of the procedure generating aerosols in newborns and supersedes all previous versions of the BAPM COVID-19 FAQ and other RCPCH guidelines. |
| (95) | Salvatore et al.  (2020) | USA | Research article | To provide guideline follow-up of neonates born to mothers who were positive for SARS-CoV-2 at delivery |
| (96) | Sankar et al.  (2020) | India | Research article | To identify COVID-19 in Children: Clinical Approach and Management- Correspondence |
| (97) | Sarman and Tuncay  (2020) | Turkey | Review article | To analyse the physical and mental health impacts of children and parents in PICU and NICU due to COVID-19 |
| (98) | Sattar and Kuperman  (2020) | USA | Review article | To discuss practical treatments for pediatric epilepsy during the COVID-19 pandemic |
| (99) | Schlaudecker  (2020) | USA | Letters to the editor | To explain essential family caregivers in long-term care during the COVID-19 pandemic |
| (100) | Schmitt and Offit  (2020) | USA | News | To discuss of fever in COVID-19 |
| (101) | Shang et al.  (2020) | China | Review article | To assist synthesis of evidence and experts’ consensus on critical care, despite the lack of clinical trials |
| (102) | Sharafi et al.  (2020) | Iran | Review article | To inform environmental disinfection against COVID-19 in different areas of health care facilities: a review |
| (103) | Shen et al.  (2020a) | China | Review article | To inform consensus statement on diagnosis, treatment and prevention of COVID-19 in children |
| (104) | Shen et al.  (2020b) | China | Review article | To analyse the latest diagnosis, treatment and prevention of COVID-19 in children. consensus of experts (condensed version of the second edition) |
| (105) | Shi et al.  (2020) | China | Observational | To describe screening of paediatric patients with suspected and confirmed COVID-19 |
| (106) | Naranje et al.  (2020) | India | Review article | To provide information management of COVID-19 in neonates |
| (107) | Skarsgard et al.  (2020) | Canada | Commentary | To describe the importance of protocols and policies for paediatric surgery |
| (108) | Slone et al.  (2020) | USA | Commentary | To provide implications of caring for children with cancer during the COVID-19 pandemic |
| (109) | Soma et al.  (2020) | Australia | Guidance | To provide guideline operational checklist for aerosol-generating procedures to minimize HCW exposure to SARS-CoV-2 |
| (110) | Sullivan et al.  (2020) | UK | Special report | To summarize general principles for continuing multidisciplinary care during the SARS-CoV-2 (COVID-19) pandemic |
| (111) | Tan et al.  (2020) | China | Original article | To develop an emergency training program of personal protective equipment (PPE) for general healthcare workers (HCWs) who may be under the threat of Corona Virus Disease 2019 (COVID-19) and evaluate the effect of the program. |
| (112) | Taylor et al.  (2020) | USA | Expert consensus | To provide innovative forms of adaptation in paediatric pulmonology during the COVID 19 pandemic |
| (113) | Teoh et al.  (2020) | Canada | Rapid communication | To inform the guideline on management of paediatric kidney transplant patients during the COVID-19 pandemic |
| (114) | Thampi et al.  (2020) | Singapore | Special interest article | To explain considerations for managing children with COVID-19 in the PICU |
| (115) | The Hastings Center  (2020) | - | Web page | To develop guidance of FCC in neonate |
| (116) | Thom et al.  (2020) | USA | Clinical overview | To analyse emergency airway management in children's hospitals before and during the COVID-19 pandemic |
| (117) | Trevisanuto et al.  (2020) | UK | Special interest article | To inform management of mothers and neonates in low resources setting during COVID-19 pandemic. |
| (118) | Tscherning et al.  (2020) | France | Clinical overview | To promote strategic attachment between parents and neonates during the COVID-19 pandemic |
| (119) | UNICEF  (2020) | - | Manual practice | To provide parents, caregivers, support persons, and children and adolescent themselves, a tool that will enable them to understand what is COVID-19 and how it can be prevented, help them Manage related stress, fear and anxiety, and recognize the increased risk of violence, which can help to them to stay safe |
| (120) | Venturini et al.  (2020) | Italy | Review article | To suggest paediatric treatment strategies for COVID-19 by the Steering and Scientific Committee of the Italian Society of Infectious Paediatric Diseases in connection with the Italian Society of Paediatrics |
| (121) | Verma et al.  (2020) | USA | Guidance | To describe preparedness of the neonatal intensive care unit for the Novel Coronavirus Disease-2019 pandemic |
| (122) | Vogt et al.  (2020) | USA | Special report | To describe provision of Paediatric Immunization Services During the COVID-19 Pandemic |
| (123) | Wang H et al.  (2020) | China | Review article | To standardize the holistic care for patients with severe coronavirus disease 2019 (COVID-19) |
| (124) | Wang J et al.  (2020) | China | Review article | To inform management of the 2019 new corona virus outbreak in the neonatal intensive care unit |
| (125) | Wang L et al.  (2020) | China | Expert consensus | To discuss prevention and control of COVID-19 infection |
| (126) | Weaver and Wiener  (2020) | USA | Expert article | To describe applying Principles of Palliative Care to Communicating with Children About COVID-19 |
| (127) | Wei et al.  (2020) | China | Short report | To investigate the environmental contamination of SARS-COV-2 by COVID-19 patients with prolonged PCR positive status of clinical samples. |
| (128) | WHO  (2020) | - | Guidance | To inform slow and stop transmission; provide optimized care for all patients; and minimize the impact of the epidemic on health systems, social services and economic activity. |
| (129) | WIDEX  (2020) | Denmark | Web page | To find out how Widex hearing aids can still be effective - and what you can do to help your clients. |
| (130) | Willer et al.  (2020) | USA | Expert article | To share experience of a large quaternary paediatric centre with COVID-19 preparation and simulation |
| (131) | Ye  (2020) | USA | Opinion | To analyse of mental health and behavioural solutions for children during quarantine and social distancing during the COVID-19 pandemic |
| (132) | Yonker et al.  (2020) | Massachusetts | Original article | To inform understanding the potential role children play in the coronavirus infectious disease 2019 (COVID-19) pandemic and the factors that drive severe illness in children is critical. |
| (133) | Zhang et al.  (2020) | Wuhan China | Review article | To develop the expert consensus on nurse's human caring for Corona Virus Disease 2019 (COVID-19) patients in different sites, and thus provide a guideline on providing whole process and systematic caring for COVID-19 patients. |
| (134) | Zimmermann and Curtis  (2020) | Australia | Special article | To share epidemiologic, clinical, and diagnostic findings, as well as treatment and prevention options for common circulating and novel CoVs infections in humans with a focus on infections in children |

ALL: Acute Leukaemia Lymphoblastic, ADHD: Attention Deficit Hyperactivity Disorder, BAPM: British Association of Perinatal Medicine, COVID-19: Coronavirus Disease-2019, CoVs: Coronaviruses, EBP: Evidence Based Practice, ED: Emergency Departments, FAQ: Frequently asked questions, FCC: Family Centred Care, PCR: Polymerase Chain Reaction, PED: Paediatric Emergency Departments, PICU: Paediatric Intensive Care Unit, RCPCH: Royal College of Paediatrics and Child Health, SARS-CoV-2: Severe Acute Respiratory Syndrome Coronavirus 2

**Reference**

1. Abuali M, Bonner R, Irigoyen M. Operationalizing an academic pediatric practice during the COVID-19 crisis. Am J Infect Control. 2021;49(2):226–8.

2. Ahmad MDF, Wahab S, Ali Ahmad F, Intakhab Alam M, Ather H, Siddiqua A, et al. A novel perspective approach to explore pros and cons of face mask in prevention the spread of SARS-CoV-2 and other pathogens. Saudi Pharm J. 2020 Dec;

3. Al Juhani T, Al Zughaibi N, Haroun A, Al Saad A. Perioperative care of pediatric anesthesia for children with suspected or confirmed COVID-19. Saudi J Anaesth. 2020;14(3):370–7.

4. American Academy of Pediatrics. FAQs: Management of Infants Born to Mothers with Suspected or Confirmed COVID-19. 2020.

5. Ashokka B, Loh MH, Tan CH, Su LL, Young BE, Lye DC, et al. Care of the pregnant woman with coronavirus disease 2019 in labor and delivery: anesthesia, emergency cesarean delivery, differential diagnosis in the acutely ill parturient, care of the newborn, and protection of the healthcare personnel. Am J Obstet Gynecol [Internet]. 2020;223(1):66-74.e3. Available from: https://doi.org/10.1016/j.ajog.2020.04.005

6. Atout M, Al-Tarawneh FS, Al-Kharabsheh A. Challenges faced by mothers caring for children with leukaemia during COVID-19 pandemic: A qualitative study. J Pediatr Nurs. 2021;

7. Aubey J, Zork N, Sheen J-J. Inpatient obstetric management of COVID-19. Semin Perinatol. 2020;44(7):151280.

8. Barney A, Buckelew S, Mesheriakova V, Raymond-Flesch M. The COVID-19 Pandemic and Rapid Implementation of Adolescent and Young Adult Telemedicine: Challenges and Opportunities for Innovation. J Adolesc Heal. 2020;67(2):164–71.

9. Udrea DS, Lopez M, Avesar M, Qureshi S, Moretti A, Abd-Allah SA, et al. Acute COVID-19 infection in a pediatric patient with ROHHAD. J Pediatr Genet. 2020;1–4.

10. Boelig RC, Manuck T, Oliver EA, Di Mascio D, Saccone G, Bellussi F, et al. Labor and delivery guidance for COVID-19. Am J Obstet Gynecol MFM. 2020;2(2, Supplement):100110.

11. Bogiatzopoulou A, Mayberry H, Hawcutt DB, Whittaker E, Munro A, Roland D, et al. COVID-19 in children: what did we learn from the first wave? Paediatr Child Health (Oxford). 2020;30(12):438–43.

12. Bouffet E, Challinor J, Sullivan M, Biondi A, Rodriguez‐Galindo C, Pritchard‐Jones K. Early advice on managing children with cancer during the COVID‐19 pandemic and a call for sharing experiences. Pediatr Blood Cancer. 2020 Apr;

13. Bressan S, Buonsenso D, Farrugia R, Parri N, Oostenbrink R, Titomanlio L, et al. Preparedness and Response to Pediatric COVID-19 in European Emergency Departments: A Survey of the REPEM and PERUKI Networks. Ann Emerg Med. 2020;76(6):788–800.

14. Campagnaro R, Collet G de O, Andrade MP de, Salles JP da SL, Calvo Fracasso M de L, Scheffel DLS, et al. COVID-19 pandemic and pediatric dentistry: Fear, eating habits and parent’s oral health perceptions. Child Youth Serv Rev. 2020;118:105469.

15. Carlotti P, Carvalho AP de, Carvalho D, Brunow W, Johnston, Rodriguez C, et al. Covid-19 diagnostic and management protocol for pediatric patients. Clinics. 2020;75:1–5.

16. Castillero AB, Beam KS, Bernardini LB, Ramos EGC, Davenport PE, Duncan AR, et al. COVID-19: neonatal–perinatal perspectives. J Perinatol. 2020;2.

17. Cavicchiolo ME, Doglioni N, Ventola MA, Biban P, Baraldi E, Trevisanuto D. Neonatal emergency transport system during COVID-19 pandemic in the Veneto Region: proposal for standard operating procedures. Pediatr Res. 2020;(April).

18. Center for Disease Control & Prevention. Strategies for Optimizing the Supply of N95 Respirators. Coronavirus 2019 (COVID-19). 2020.

19. Chawla D, Chirla D, Dalwai S, Deorari AK, Ganatra A, Gandhi A, et al. Perinatal-neonatal Management of COVID-19 infection — Guidelines of the Federation of Obstetric and Gynaecological Societies of India (FOGSI), National Neonatology Forum of India (NNF), and Indian Academy of Pediatrics (IAP). Indian Pediatr. 2020;57(6):536–48.

20. Chen H, Selix N, Nosek M. Perinatal Anxiety and Depression During COVID-19. J Nurse Pract. 2021;17(1):26–31.

21. Chen ZM, Fu JF, Shu Q, Chen YH, Hua CZ, Li FB, et al. Diagnosis and treatment recommendations for pediatric respiratory infection caused by the 2019 novel coronavirus. World J Pediatr. 2020;16(3):240–6.

22. Choi KR, Records K, Low LK, Alhusen JL, Kenner C, Bloch JR, et al. Promotion of maternal–infant mental health and trauma-informed care during the COVID-19 pandemic. J Obstet Gynecol Neonatal Nurs [Internet]. 2020;49(5):409–15. Available from: http://www.sciencedirect.com/science/article/pii/S0884217520301155

23. DeFazio JR, Kahan A, Fallon EM, Griggs C, Kabagambe S, Zitsman J, et al. Development of pediatric surgical decision-making guidelines for COVID-19 in a New York City children’s hospital. J Pediatr Surg. 2020;55(8):1427–30.

24. Aleebrahim-Dehkordi E, Soveyzi F, Deravi N, Rabbani Z, Saghazadeh A, Rezaei N. Human coronaviruses SARS-CoV, MERS-CoV, and SARS-CoV-2 in children. J Pediatr Nurs [Internet]. 2020;56:70–9. Available from: https://doi.org/10.1016/j.pedn.2020.10.020

25. Deitrick K, Adams J, Davis J. Emergency Nursing Care of Patients With Novel Coronavirus Disease 2019. J Emerg Nurs. 2020;46(6):748–59.

26. Deprest J, Choolani M, Chervenak F, Farmer D, Lagrou K, Lopriore E, et al. Fetal diagnosis and therapy during the COVID-19 pandemic: Guidance on behalf of the International Fetal Medicine and Surgery Society. Fetal Diagn Ther. 2020 May;1–10.

27. Devrim I, Bayram N. Infection control practices in children during COVID-19 pandemic : Differences from adults. Am J Infect Control. 2020;00:1–7.

28. Ding H, Shi Z, Ruan Z, Cheng X, Li R, Zhang L, et al. Consideration of the Management of Pediatric Fever Clinics During the Novel Coronavirus Pneumonia Outbreak. Disaster Medicine and Public Health Preparedness. Cambridge: Cambridge University Press; 2020 Aug.

29. Dokken D, Ahmann E, Miller D, Weaver J. Mental health needs during COVID-19: Responses in pediatric health care. Pediatr Nurs [Internet]. 2020 Nov;46(6):304–7. Available from: http://search.ebscohost.com/login.aspx?direct=true&db=rzh&AN=147618061&site=ehost-live

30. Dumpa V, Kamity R, Vinci AN, Noyola E, Noor A. Neonatal Coronavirus 2019 (COVID-19) infection: A case report and review of literature. Cureus. 2020;2019(5).

31. ELSO. Extracorporeal Life Support Organization COVID-19 Interim Guidelines. ELSO Gen Guidel. 2020;37.

32. Enyama D, Chelo D, Njinkui DN, Kouam JM, Puepi YFD, Nkwele IM, et al. Impact of the COVID-19 pandemic on pediatricians’ clinical activity in Cameroon. Arch Pediatr. 2020;27(8):423–7.

33. ESPNIC. Nursing guidance for the care of the child with suspected or proven COVID-19 infection. European Society for Pediatric and Neonatal Intensive Care; 2020. p. 1–2.

34. Evans Y, Golub S, Sequeira GM, Eisenstein E, North S. Using telemedicine to reach adolescents during the COVID-19 pandemic. 2020;(January).

35. Ezenwa BN, Fajolu IB, Akinajo OR, Makwe CC, Oluwole AA, Akase IE, et al. Management of covid-19: a practical guideline for maternal and newborn health care providers in Sub-Saharan Africa. J Matern Neonatal Med [Internet]. 2020;0(0):1–7. Available from: https://doi.org/10.1080/14767058.2020.1763948

36. Farshi MR, Jabraeili M, Moharrami N, Malakouti J. Nursing care in a child with Coronavirus Disease 2019: A case study. Hormozgan Med J. 2020;24(4):e108040.

37. Frauenfelder C, Butler C, Hartley B, Cochrane L, Jephson C, Nash R, et al. Practical insights for paediatric otolaryngology surgical cases and performing microlaryngobronchoscopy during the COVID-19 pandemic. Int J Pediatr Otorhinolaryngol. 2020;134:1–4.

38. Gale C, Quigley MA, Placzek A, Knight M, Ladhani S, Draper ES, et al. Characteristics and outcomes of neonatal SARS-CoV-2 infection in the UK: A prospective national cohort study using active surveillance. Lancet Child Adolesc Heal. 2021;5(2):113–21.

39. Gerall CD, DeFazio JR, Kahan AM, Fan W, Fallon EM, Middlesworth W, et al. Delayed presentation and sub-optimal outcomes of pediatric patients with acute appendicitis during the COVID-19 pandemic. J Pediatr Surg. 2020;

40. Giuliani C, Li Volsi P, Brun E, Chiambretti A, Giandalia A, Tonutti L, et al. Breastfeeding during the COVID-19 pandemic: Suggestions on behalf of woman study group of AMD. Diabetes Res Clin Pract [Internet]. 2020;165:108239. Available from: https://doi.org/10.1016/j.diabres.2020.108239

41. Gralton KS, Korom N, Kavanaugh K, Wenner S, Norr K. COVID-19: Impact for pediatric research, evidence-based practice and quality processes and projects. J Pediatr Nurs. 2020;55:264–5.

42. Green J, Petty J, Bromley P, Walker K, Jones L. COVID-19 in babies: Knowledge for neonatal care. J Neonatal Nurs [Internet]. 2020;26(5):239–46. Available from: http://www.sciencedirect.com/science/article/pii/S135518412030096X

43. Green J, Petty J, Whiting L, Fowler C. Exploring modifiable risk-factors for premature birth in the context of COVID-19 mitigation measures: A discussion paper. J Neonatal Nurs. 2020;

44. Green J, Staff L, Bromley P, Jones L, Petty J. The implications of face masks for babies and families during the COVID-19 pandemic: A discussion paper. J Neonatal Nurs [Internet]. 2021;27:21–5. Available from: https://doi.org/10.1016/j.jnn.2020.10.005

45. Greene NH, Kilpatrick SJ, Wong MS, Ozimek JA, Naqvi M. Impact of labor and delivery unit policy modifications on maternal and neonatal outcomes during the coronavirus disease 2019 pandemic. Am J Obstet Gynecol MFM. 2020;2(4, Supplement):100234.

46. Hahn A, Vater L. COVID-19 and kids: Managing chronic pain at home. Nationwide Children’s Hospital. 2020.

47. Hanekamp C, Huang FS. COVID-19 (Coronavirus) [Internet]. 2020 [cited 2021 Jan 23]. Available from: https://www.cincinnatichildrens.org/health/c/covid-19-coronavirus

48. Hart JL, Turnbull AE, Oppenheim IM, Courtright KR. Family-Centered Care during the COVID-19 era. J Pain Symptom Manage [Internet]. 2020;60(2):e93–7. Available from: https://doi.org/10.1016/j.jpainsymman.2020.04.017

49. Hester M. AAP issues guidance for managing infants born to mothers with COVID-19. Contemp Pediatr. 2020 Jul;37(7):23.

50. Hester M. A discussion on ADHD in the COVID era. Contemp Pediatr. 2021 Jan;38(1):19.

51. Houtrow A, Harris D, Molinero A, Levin-Decanini T, Robichaud C. Children with disabilities in the United States and the COVID-19 pandemic. J Pediatr Rehabil Med. 2020;10(36):1–10.

52. Human Milk Banking Association of North America. Milk Handling for COVID-19 Positive or Suspected Mothers in the Hospital Setting References. 2020;31(2010):2019–20.

53. Ingram M-CE, Raval M V, Newton C, Lopez ME, Berman L. Characterization of initial north American pediatric surgical response to the COVID-19 pandemic. J Pediatr Surg. 2020;55(8):1431–5.

54. Jain PN, Finger L, Schieffelin JS, Zerr DM, Hametz PA. Responses of three urban U.S. Children’s Hospitals to COVID-19: Seattle, New York and New Orleans. Paediatr Respir Rev. 2020;35:15–9.

55. Jiang L, Tang K, Levin M, Irfan O, Morris SK, Wilson K, et al. COVID-19 and multisystem inflammatory syndrome in children and adolescents. Lancet Infect Dis. 2020;20(11):e276–88.

56. Jiao WY, Wang LN, Liu J, Fang SF, Jiao FY, Pettoello-Mantovani M, et al. Behavioral and Emotional Disorders in Children during the COVID-19 Epidemic. Vol. 221, Journal of Pediatrics. 2020. p. 264-266.e1.

57. Johnson SJ, Dupler AE. Pediatric Surgery Nurses Lead and Coordinate COVID-19 Recovery Efforts. Pediatr Nurs. 2020 Sep;46(5):219–23.

58. Joosten KFM, Eveleens RD, Verbruggen SCAT. Nutritional support in the recovery phase of critically ill children. Curr Opin Clin Nutr Metab Care. 2019;22(2):152–8.

59. Kache S, Chisti MJ, Gumbo F, Mupere E, Zhi X, Nallasamy K, et al. COVID-19 PICU guidelines: for high- and limited-resource settings. Pediatr Res. 2020;(April).

60. Kallem VR, Sharma D. COVID 19 in neonates. J Matern Neonatal Med. 2020;1–9.

61. Kari JA, Shalaby MA, Albanna AS, Alahmadi T, Sukkar SA, MohamedNur HAH, et al. Coronavirus Disease in Children: A Multicentre Study from the Kingdom of Saudi Arabia. J Infect Public Health. 2021;

62. Karimi A, Tabatabaei, S. R Rajabnejad, M Pourmoghaddas, Z Rahimi H, Armin S, Ghanaie RM, Kadivar MR, Fahimzad SA, et al. An algorithmic approach to diagnosis and treatment of coronavirus disease 2019 (COVID-19) in children: Iranian expert’s consensus statement. Arch Pediatr Infect Dis. 2020;8(2).

63. Kassab M, Nuseair K, Al-Qaoud N, Hamadneh S, Roy AN. Is facilitated tucking by parents more effective than dextrose water (10%) in reducing full-term neonatal pain during the heel-lancing procedure: A randomized controlled trial. Int J Pharm Res. 2020;12(1):2178–86.

64. Kaushik A, Gupta S, Sood M. COVID-19 in Children: Clinical Approach and Management- Correspondence. Indian J Pediatr. 2020;87:433–42.

65. Kazzaz Y m, Alkhalaf H, Alharbi M, Al Shaalan M, Almuneef M, Alshehri A, et al. Hospital preparedness and management of pediatric population during COVID‑19 outbreak. Ann Thorac Meedicine. 2020;15(3):107–17.

66. Kneyber MCJ, Medina A, Alapont VM i, Brierly J, Chidini G, Cusco MG, et al. Practice recommendations for the management of children with suspected or proven COVID-19 infections from the Paediatric Mechanical Ventilation Consensus Conference ( PEMVECC ) and the section Respiratory Failure from the European Society for Paediatric a. Eur Soc Pediatr Neonatal Intensive Care. 2020;1–7.

67. Krishnamurthy G, Sahni R, Leone T, Kim F, Brooks MC, Morales SV, et al. Care of the COVID-19 exposed complex newborn infant. Semin Perinatol. 2020;44(7):151282.

68. Kumar P, Dawman L, Panda P, Kumar I. Feasibility and effectiveness of teleconsultation in children with epilepsy amidst the ongoing COVID-19 pandemic in a resource-limited country. Seizure Eur J Epilepsy. 2020;(January):29–35.

69. Lakhani A, Sharma E. Corona virus (Covid-19) – ITS implications in pediatric orthopedic care. J Orthop. 2020;21:326–30.

70. Leff RA, Setzer E, Cicero MX, Auerbach M. Changes in pediatric emergency department visits for mental health during the COVID-19 pandemic: A cross-sectional study. Clin Child Psychol Psychiatry. 2020;1–6.

71. López-Bueno R, López-Sánchez GF, Casajús JA, Calatayud J, Tully MA, Smith L. Potential health-related behaviors for pre-school and school-aged children during COVID-19 lockdown: A narrative review. Prev Med (Baltim). 2021;143:106349.

72. Lopez AS, Hill M, Antezano J, Vilven D, Rutner T, Bogdanow L, et al. Transmission dynamics of COVID-19 outbreaks associated with child care facilities - Salt Lake City, Utah, April-July 2020. MMWR Morb Mortal Wkly Rep [Internet]. 2020 Sep 18;69(37):1319–23. Available from: http://search.ebscohost.com/login.aspx?direct=true&db=rzh&AN=145926260&site=ehost-live

73. Lotfi M, Hamblin MR, Rezai nima. COVID-19: Transmission, prevention, and potential therapeutic opportunities. Clin Chim Acta. 2020;508:254–66.

74. MaGowan N, Darcy J, Mosiello A, Gomes C, Miller N. Navigating Through the Uncharted Territory of Multisystem Inflammatory Syndrome in Children (MIS-C): What the Pediatric Clinical Nurse Must Knowtle. Pediatr Nurs. 2020;46(6):273–7.

75. Marino L., Valla F., Turne L., Jotterand-Chaparro C, Moullet C, Latten L, et al. Considerations for nutrition support in critically ill children with COVID-19 and paediatric inflammatory multisystem syndrome temporally associated with COVID-19. 2020;(January).

76. Marraro GA, Spada C. Consideration of the respiratory support strategy of severe acute respiratory failure caused by SARS-CoV-2 infection in children. Chinese J Contemp Pediatr. 2020;22(3):183–94.

77. Matava CT, Kovatsis PG, Lee JK, Castro P, Denning S, Yu J, et al. Pediatric airway management in COVID-19 patients: Consensus guidelines from the Society for Pediatric Anesthesia’s Pediatric Difficult Intubation Collaborative and the Canadian Pediatric Anesthesia Society. Anesth Analg. 2020;131(1):61–73.

78. Michigan Department of Health & Human Services (MDHHS). COVID-19 Guidance for Healthcare Facilities for Discharge of Residents. 2020;

79. Mihatsch W, Shamir R, van Goudoever JB, Fewtrell M, Lapillonne A, Lohner S, et al. ESPGHAN/ESPEN/ESPR/CSPEN guidelines on pediatric parenteral nutrition: Guideline development process for the updated guidelines. Clin Nutr. 2018;37(6):2306–8.

80. Mirlashari J, Ebrahimpour F, Salisu WJ. War on two fronts: Experience of children with cancer and their family during COVID-19 pandemic in Iran. J Pediatr Nurs. 2021;57:25–31.

81. Montes MT, Herranz-Rubia N, Ferrero A, Flórez A, Quiroga A, Gómez A, et al. Neonatal nursing in the COVID-19 pandemic: can we improve the future? J Neonatal Nurs. 2020;26(5):247–51.

82. Moro GE, Bertino E. Breastfeeding, human milk collection and containers, and human milk banking: Hot topics during the COVID-19 pandemic. J Hum Lact. 2020;36(4):604–8.

83. Monzani A, Ragazzoni L, Della Corte F, Rabbone I, Franc JM. COVID-19 Pandemic: Perspective from Italian Pediatric Emergency Physicians. Disaster Med Public Health Prep. 2020;14(5):648–51.

84. Mulay KV, Aishworiya R, Lim TSH, Tan MY, Kiing JSH, Chong SC, et al. Innovations in practice: Adaptation of developmental and behavioral pediatric service in a tertiary center in Singapore during the COVID-19 pandemic. Pediatr Neonatol. 2021;62(1):70–9.

85. Nicholas DB, Belletrutti M, Dimitropoulos G, Katz SL, Rapoport A, Urschel S, et al. Perceived Impacts of the COVID-19 Pandemic on Pediatric Care in Canada: A Roundtable Discussion. Glob Pediatr Heal. 2020;7.

86. Nolana JP, Monsieursc KG, Bossaertd L, Bottigerf BW, Greifg R, Lotth C, et al. European Resuscitation Council COVID-19 guidelines executive summary. Resuscitation. 2020;153:45–55.

87. Peck JL. COVID-19: Impacts and Implications for Pediatric Practice. J Pediatr Heal Care. 2020;34(6):619–29.

88. Peng S, Zhu H, Yang L, Cao L, Huang X, Dynes M, et al. A study of breastfeeding practices, SARS-CoV-2 and its antibodies in the breast milk of mothers confirmed with COVID-19. Lancet Reg Heal - West Pacific. 2020;4:100045.

89. Pereira A, Cruz-Melguizo S, Adrien M, Fuentes L, Marin E, Forti A, et al. Breastfeeding mothers with COVID-19 infection: A case series. Int Breastfeed J. 2020;15(69):1–8.

90. Rajapakse N, Dixit D. Human and novel coronavirus infections in children: a review. Paediatr Int Child Health. 2020;00(00):1–20.

91. Rathore P, Kumar S, Shweta, Singh N, Krishnapriya V, Thankachan A, et al. A Child with Acute Lymphoblastic Leukemia in Institutional Isolation during the COVID Pandemic: A Multifaceted Responsibility. Indian J Palliat Care. 2020;26(5):S170–2.

92. Robinson C, Ruhl M, Kirpalani A, Alabbas A, Noone D, Teoh CW, et al. Management of Canadian pediatric patients with glomerular diseases during the COVID-19 pandemic: Recommendations from the Canadian Association of Pediatric Nephrologists COVID-19 Rapid Response Team. Can J Kidney Heal Dis. 2020;7:1–17.

93. Rodríguez Yago MA, Alcalde Mayayo I, Gómez López R, Parias Ángel MN, Pérez Miranda A, Canals Aracil M, et al. Recommendations on cardiopulmonary resuscitation in patients with suspected or confirmed SARS-CoV-2 infection (COVID-19). Executive summary. Med Intensiva (English Ed. 2020;44(9):566–76.

94. Royal College of Paediatrics and Child Health. BAPM - COVID-19: Frequently asked questions within neonatal services. Br Assoc Perinat Med. 2020;

95. Salvatore CM, Han J-Y, Acker KP, Tiwari P, Jin J, Brandler M, et al. Neonatal management and outcomes during the COVID-19 pandemic: an observation cohort study. Lancet Child Adolesc Heal. 2020;4(10):721–7.

96. Sankar J, Dhochak NS, Kabra S., Lodha R. COVID-19 in Children: Clinical Approach and Management- Correspondence. Indian J Pediatr. 2020;87(11):970–2.

97. Sarman A, Tuncay S. Principles of approach to suspected or infected patients related Covid‐19 in newborn intensive care unit and pediatric intensive care unit. Perspect Psychiatr Care [Internet]. 2020 Nov 13;57:957–964. Available from: https://search.proquest.com/scholarly-journals/principles-approach-suspected-infected-patients/docview/2460222340/se-2?accountid=17242

98. Sattar S, Kuperman R. Telehealth in pediatric epilepsy care: A rapid transition during the COVID-19 pandemic. Epilepsy Behav. 2020;111:107282.

99. Schlaudecker JD. Essential family caregivers in long-term care during the COVID-19 pandemic. J Am Med Dir Assoc [Internet]. 2020;21(7):983–4. Available from: https://doi.org/10.1016/j.jamda.2020.05.027

100. Schmitt BD, Offit PA. Could fever improve COVID-19 outcomes? Vol. 37, Contemporary Pediatrics. MJH Life Sciences; 2020. p. 6–33.

101. Shang Y, Pan C, Yang X, Zhong M, Shang X, Wu Z, et al. Management of critically ill patients with COVID-19 in ICU: statement from front-line intensive care experts in Wuhan, China. Ann Intensive Care. 2020;10(1):1–24.

102. Sharafi SM, Ebrahimpour K, Nafez A. Environmental disinfection against COVID-19 in different areas of health care facilities: A review. Rev Environ Health. 2020;

103. Shen KL, Yang YH, Jiang RM, Wang TY, Zhao DC, Jiang Y, et al. Updated diagnosis, treatment and prevention of COVID-19 in children: experts’ consensus statement (condensed version of the second edition). Vol. 16, World Journal of Pediatrics. 2020. p. 232–9.

104. Shen K, Yang Y, Wang T, Zhao D, Jiang Y, Jin R, et al. Diagnosis, treatment, and prevention of 2019 novel coronavirus infection in children: experts’ consensus statement. World J Pediatr. 2020;16(3):223–31.

105. Shi Y, Wang X, Liu G, Zhu Q, Wang J, Yu H, et al. A quickly, effectively screening process of novel corona virus disease 2019 (COVID-19) in children in Shanghai, China. Ann Transl Med. 2020 Mar;8(5):241–241.

106. Naranje KM, Gupta G, Singh A, Bajpai S, Verma A, Jaiswal R, et al. Neonatal COVID-19 Infection Management. J Neonatol. 2020;34(1–2):88–98.

107. Skarsgard ED, Skarsgard ED, Bass J, Beaudry P, Corsten G, Drake J, et al. Prioritizing specialized children’s surgery in Canada during the COVID-19 pandemic. Cmaj. 2020;192(41):E1212–3.

108. Slone JS, Ozuah N, Wasswa P. Caring for children with cancer in Africa during the COVID-19 crisis: Implications and opportunities. Pediatr Hematol Oncol [Internet]. 2020;37(7):549–53. Available from: https://doi.org/10.1080/08880018.2020.1772913

109. Soma M, Jacobson I, Brewer J, Blondin A, Davidson G, Singham S. Operative team checklist for aerosol generating procedures to minimise exposure of healthcare workers to SARS-CoV-2. Int J Pediatr Otorhinolaryngol. 2020;134:110075.

110. Sullivan M, Bouffet E, Rodriguez-Galindo C, Luna-Fineman S, Khan MS, Kearns P, et al. The COVID-19 pandemic: A rapid global response for children with cancer from SIOP, COG, SIOP-E, SIOP-PODC, IPSO, PROS, CCI, and St Jude Global. Pediatr Blood Cancer. 2020;67(7):1–12.

111. Tan W, Ye Y, Yang Y, Chen Z, Yang X, Zhu C, et al. Whole-Process Emergency Training of Personal Protective Equipment Helps Healthcare Workers against COVID-19: Design and Effect. J Occup Environ Med. 2020;62(6):420–3.

112. Taylor JB, Oermann CM, Deterding RR, Redding G, Davis SD, Piccione J, et al. Innovating and Adapting in Pediatric Pulmonology and Sleep Medicine During the COVID‐19 Pandemic: ATS Pediatric Assembly Web Committee Consensus Statement for Initial COVID‐19 Virtual Response. Pediatr Pulmonol. 2020 Dec;

113. Teoh CW, Gaudreault-Tremblay MM, Blydt-Hansen TD, Goldberg A, Arora S, Feber J, et al. Management of Pediatric Kidney Transplant Patients During the COVID-19 Pandemic: Guidance From the Canadian Society of Transplantation Pediatric Group. Can J Kidney Heal Dis. 2020;7.

114. Thampi S, Yap A, Fan L, Ong J. Special considerations for the management of COVID-19 pediatric patients in the operating room and pediatric intensive care unit in a tertiary hospital in Singapore. Paediatr Anaesth. 2020;1–5.

115. The Hastings Center. Should new mothers with Covid-19 be separated from their newborns? 2020.

116. Thom CS, Deshmukh H, Soorikian L, Jacobs I, Fiadjoe JE, Lioy J. Airway emergency management in a pediatric hospital before and during the COVID-19 pandemic. Int J Pediatr Otorhinolaryngol. 2020;139(October):110458.

117. Trevisanuto D, Weiner G, Lakshminrusimha S, Azzimonti G, Nsubuga JB, Velaphi S, et al. Management of mothers and neonates in low resources setting during covid-19 pandemia. J Matern Neonatal Med [Internet]. 2020 Jun 30;1–12. Available from: https://doi.org/10.1080/14767058.2020.1784873

118. Tscherning C, Sizun J, Kuhn P. Promoting attachment between parents and neonates despite the COVID-19 pandemic. Acta Paediatr Int J Paediatr. 2020;109(10):1937–43.

119. UNICEF. Psychosocial Support for Children during COVID-19. Child Line India Foundation. 2020. p. 2–5.

120. Venturini E, Montagnani C, Garazzino S, Donà D, Pierantoni L, Lo Vecchio A, et al. Treatment of children with COVID-19: position paper of the Italian Society of Pediatric Infectious Disease. Ital J Pediatr. 2020;46(1):139.

121. Verma S, Lumba R, Lighter JL, Bailey SM, Wachtel E V, Kunjumon B, et al. Neonatal intensive care unit preparedness for the Novel Coronavirus Disease-2019 pandemic: A New York City hospital perspective. Curr Probl Pediatr Adolesc Health Care. 2020;50(4):100795.

122. Vogt TM, Zhang F, Banks M, Black C, Arthur B, Kang Y, et al. Provision of Pediatric Immunization Services During the COVID-19 Pandemic: an Assessment of Capacity Among Pediatric Immunization Providers Participating in the Vaccines for Children Program - United States, May 2020. MMWR Morb Mortal Wkly Rep. 2020 Jul;69(27):859–63.

123. Wang H, Zeng T, Wu X, Sun H. Holistic care for patients with severe coronavirus disease 2019: An expert consensus. Int J Nurs Sci. 2020;7(2):128–34.

124. Wang J, Qi H, Bao L, Li F, Shi Y. A contingency plan for the management of the 2019 novel coronavirus outbreak in neonatal intensive care units. Lancet Child Adolesc Heal. 2020;4(4):258–9.

125. Wang L, Shi Y, Xiao T, Fu J, Feng X, Mu D, et al. Chinese expert consensus on the perinatal and neonatal management for the prevention and control of the 2019 novel coronavirus infection (First edition). Ann Transl Med. 2020;8(3):47.

126. Weaver MS, Wiener L. Applying Palliative Care Principles to Communicate With Children About COVID-19. J Pain Symptom Manage. 2020;60(1):e8–11.

127. Wei L, Huang W, Lu X, Wang Y, Cheng L, Deng R, et al. Contamination of SARS-CoV-2 in patient surroundings and on personal protective equipment in a non-ICU isolation ward for COVID-19 patients with prolonged PCR positive status. Antimicrob Resist Infect Control. 2020 Dec;9(1):167.

128. WHO. Clinical management of COVID-19: Interim guidance. WHO Media Centre; 2020. p. 1–55.

129. WIDEX. How you can help communication when face masks are needed. WIDEX Audiol. Bull. 2020. p. 85.

130. Willer BL, Thung AK, Corridore M, D’Mello AJ, Schloss BS, Malhotra PS, et al. The otolaryngologist’s and anesthesiologist’s collaborative role in a pandemic: A large quaternary pediatric center’s experience with COVID-19 preparation and simulation. Int J Pediatr Otorhinolaryngol. 2020;136:110174.

131. Ye J. Pediatric mental and behavioral health in the period of quarantine and social distancing with COVID-19. JMIR Pediatr Parent. 2020;3(2).

132. Yonker LM, Neilan AM, Bartsch Y, Patel AB, Regan J, Arya P, et al. Pediatric Severe Acute Respiratory Syndrome Coronavirus 2 (SARS-CoV-2): Clinical Presentation, Infectivity, and Immune Responses. J Pediatr. 2020;227:45-52.e5.

133. Zhang feng J, Hu DY, Liu Y-L, Li H, Zhu X-P, Pan S-S. Expert Consensus on Nurses’ Human Caring for COVID-19 Patients in Different Sites. Curr Med Sci. 2020 Aug;40(4):602–7.

134. Zimmermann P, Curtis N. Coronavirus infections in children including COVID-19. An overview of the epidemiology, clinical features, diagnosis, treatment and prevention options in children. Pediatr Infect Dis J. 2020;39(5):355–68.
